# High-resolution genomic analysis reveals abundant mosaic outcomes of bacterial natural transformation independent of MutS-mediated mismatch repair

**DOI:** 10.1128/mbio.00444-26

**Published:** 2026-06-15

**Authors:** Jonathan M. Lombardino, Tanya G. Falbel, Colin N. Dewey, Briana M. Burton

**Affiliations:** 1 Department of Bacteriology, University of Wisconsin–Madison, Madison, Wisconsin, USA; 2 Microbiology Doctoral Training Program, University of Wisconsin–Madison, Madison, Wisconsin, USA; 3 Department of Biostatistics and Medical Informatics, University of Wisconsin–Madison, Madison, Wisconsin, USA; The Ohio State University 2647, Columbus, Ohio, USA

**Keywords:** *Bacillus subtilis*, natural transformation systems, genetic competence, cyanobacteria, genome analysis

## Abstract

The nature and breadth of horizontal gene transfer outcomes specific to natural transformation remain elusive. We present a genome-scale analysis of location-specific information associated with single-round transformation events in *Bacillus subtilis*. Using distributed selectable markers to remove location bias, we found transformant genomes often contained multiple discontinuous segments of donor sequence in close proximity. These highly mosaic sites span multiple length scales, with an abundance of shorter segments. We found that the small segments scale with the length of the nearest stretch of perfect homology, and these segments defy minimal, efficient homologous recombination rules. Sites of transformation and their associated intervening recipient sequences were not distinguished by overall percent identity, GC content, or median gene expression. Mismatch repair activity by MutS also failed to explain the breadth and frequency of mosaic patches. High-resolution mapping of donor and recipient alleles across sites of transfer demonstrates that natural transformation can contribute a breadth of allelic diversity, especially within short, clustered patches of genetic exchange. These observations point to a need to further investigate the complex mechanisms that drive distinct outcomes of natural transformation.

**IMPORTANCE:**

Several works have suggested the potential for discontinuity for donor DNA in transforming DNA. This work developed robust bioinformatic and genomic approaches to assess the full breadth of exchange between divergent genomes during natural transformation. The results demonstrate that simplistic sequence and expression-based associations are not sufficient to explain highly variable transformation outcomes. Similarly, transformant genomes are frequently incongruent with previously defined rules for homology-mediated recombination. MutS-mediated mismatch repair, a frequently proposed contributor to mosaic recombination, is also insufficient to explain discontinuity. Therefore, widespread molecular mechanisms intrinsic to recombination have the potential to generate significant genetic diversity during transformation, ranging from the scale of individual alleles to full operons. These results further reinforce the role of natural transformation in shaping genetic diversity within bacterial populations.

## INTRODUCTION

Natural transformation (NT) is a widely conserved mechanism of horizontal gene transfer (HGT), spanning both gram-positive and gram-negative bacteria (1). In contrast to transduction and conjugation, NT is independent of donor-derived mobile genetic elements and therefore is not limited by defined mating pairs and phage-host relationships (2, 3). Instead, naturally transformable organisms encode DNA uptake proteins to bind, trap, and internalize DNA from their environments, which can be recombined into the recipient genome. The internalization of environmental DNA may be used for fidelity (4, 5), evolution (1, 6), or nutrients (7, 8). The benefits derived from each of these possibilities depend upon the degree of similarity between the environmental donor DNA and the recipient genome. If donor DNA originates from the same strain, the DNA can serve as a template for homologous recombination to ameliorate deleterious alleles (9), repair DNA lesions (4, 5, 10), and remove harmful mobile genetic elements (11). Conversely, environmental DNA may come from a closely related organism with novel genetic material, allowing for NT to promote evolution by acquiring new genes and alleles as a form of bacterial sexual reproduction (6, 9). However, unrelated or distantly related DNA may lack sufficient levels of similarity to initiate homologous recombination (12, 13).

Although the ancestral function of natural transformation remains debated, its impact on genome evolution is evident. Specifically, experiments in several species have demonstrated NT’s ability to confer novel adaptations that increase fitness amidst changing environments (14–18). Indeed, several *in silico* studies suggest the presence of NT machinery is correlated with higher genome diversity (19, 20). Yet, capturing direct evidence for NT in mixed bacterial populations is often challenging due to the lack of a specific sequence signature for tracking DNA exchange. Retrospective genome analyses therefore rely on algorithms to detect homologous recombination assuming large continuous transfers (21). However, since NT results in discontinuous patterns of exchange (22–24), more experimental insights are needed to understand how NT contributes to novel combinations and reassortment of alleles amidst bacterial populations. Here, we developed an analysis pipeline to explore the nucleotide signatures of interspecies NT outcomes using the model organism *Bacillus subtilis*.

Owing to the non-specific nature of DNA uptake (25–27), few restriction enzymes, and overall genetic tractability (28), *B. subtilis* has been a fruitful model for understanding the mechanisms underpinning NT (26, 29). Under nutrient-limiting conditions and the onset of stationary phase, roughly 10–20% of *B. subtilis* cells undergo transcriptional reprogramming via the ComK transcription factor, resulting in a morphologically distinct and replication-quiescent cell state called competence (30, 31). Cells in the competent state bind DNA, and through a series of steps import a transforming DNA strand into the cytosol (26, 29, 32–38). This single strand acts as a substrate for RecA nucleofilament assembly and ultimately strand exchange between the donor DNA and its homologous recipient duplex DNA (39).

While the DNA uptake by *B. subtilis* is indiscriminate, natural transformation is discriminatory with respect to the level of sequence relatedness between donor and recipient DNAs (12, 13). Indeed, assays targeting the *rpoB* locus have demonstrated that the efficiency of transformation decreases in a log-linear fashion with increasing sequence divergence between the donor and recipient DNA (12, 40, 41). Mismatch repair has been shown to influence the efficiency of transformation (41–46); however, loss-of-function mutations in the mismatch-repair enzyme *mutS* in *B. subtilis* contribute little to no influence on interspecies transformation (40, 41, 45). Yet, the broader applicability of these findings is challenged by the highly essential nature of the *rpoB* locus, as certain donor alleles may be underrepresented due to negative fitness contributions in the resulting transformant.

Whole-genome sequencing has enabled genome-scale insights into the dynamics of NT. In several competent species, the field has addressed questions, such as how large continuous transfers can be in settings of close cellular contact (47–49), how sequence variation affects outcomes of transformation (22, 50, 51), and what rate of genome replacement occurs over time through iterative rounds of transformation (14, 22, 52). Despite disparate organisms and approaches, these works are largely biased to single marker locations, or rely on non-native selective forces to assay NT. This raised the question then, of whether a comprehensive analysis of single rounds of NT uncoupled from single marker sites could disentangle sequence-level requirements for transformation (22).

Here, we overcome single-locus bias and intrinsic fitness constraints by using a distributed marker approach to analyze genome-scale transformation in *B. subtilis*. In tandem, we deploy a novel probabilistic multinomial hidden Markov model (MHMM) to confidently assign donor and recipient alleles across multiple scales of exchange and across the bacterial domain. We report that natural transformation contributes to highly dynamic lengths of transfer, spanning from less than 100 bp to over 30 kb. We demonstrate that single rounds of natural transformation generate highly mosaic patterns of transfer across the genome. Mosaic transformation was not attributable to local or global patterns of percent identity, GC content, or median measurements of gene expression. Finally, mosaic outcomes were frequent even in the absence of MutS-mediated mismatch repair.

## RESULTS

### Genome-scale approaches reveal frequent mosaic transformation events

**Fig 1:**
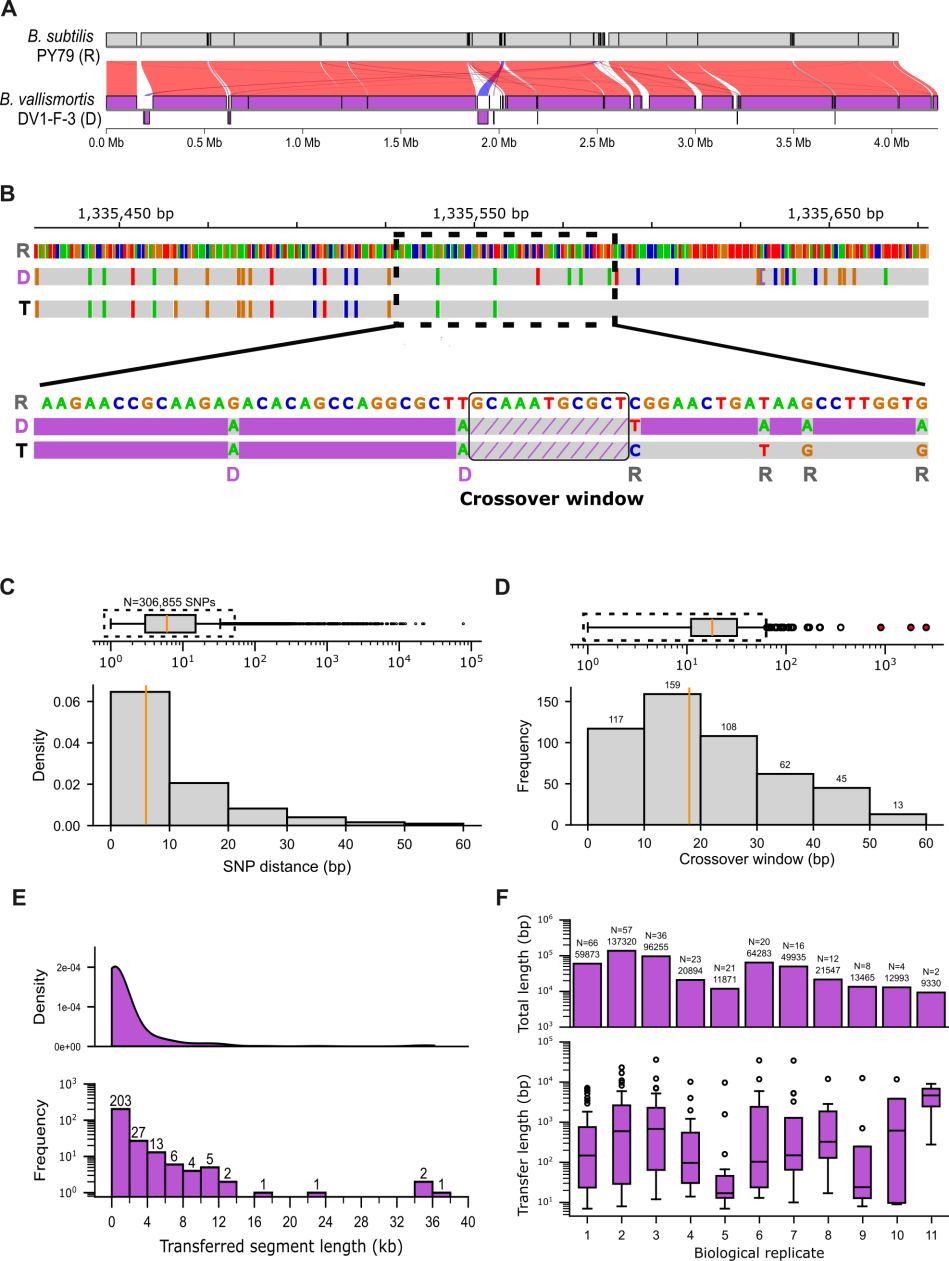
Single-round transformation of *B. subtilis* with a divergent donor reveals variable length transfer events. (**A**) Linear genome diagram of the locally collinear blocks between the naturally transformable recipient, *B. subtilis* PY79 (top), and the donor *B. vallismortis* DV1-F-3 (bottom), on the plus strand (red) or minus (blue). (**B**) A representative visualization of the ambiguity for the sites of crossing over on one side of a double-crossover event. The recipient genome sequence (top, R) is aligned to its orthologous donor sequence (middle, D) to highlight SNPs that distinguish between the two genome states, and their presence or absence in the transformant (bottom, T). The zoomed inset visualizes the crossover window, the region of identical bases situated between the donor (D) and recipient (R) distinguishing alleles, in which the exact site of crossing over cannot be precisely resolved. (**C**) The distances between SNPs from a whole-genome alignment between the donor and recipient strains as a box and whisker plot (top). (**D**) The crossover window lengths on both sides of the transferred donor DNA segments as a box and whisker plot (top) and histogram (bottom). Red outliers correspond to rRNA operon sites that were omitted from the remaining analyses. (**C and D**) The full distribution of the values is plotted in the top axis in the form of a boxplot. A subset of the non-outlier values is visualized in the form of a histogram to capture the shape of the data. (**E**) The distribution of transferred donor segment lengths in kilobases, as a kernel density estimate (top) and as a histogram visualized with a bin size of 2 kb. (**F**) The total length of base pairs transferred for each transformant (top) and the length of their individual transferred segments on a logarithmic scale (bottom) for each biological replicate is displayed.

To generate genome-scale insights into natural transformation*,* we developed a methodology that randomly distributes selectable markers throughout donor genomic DNA (gDNA) followed by transformation into a recipient, and high-resolution analysis of the transformant genomes. This method employs *in vitro* transposition using a custom Tn5 spectinomycin resistance cassette, *spcR* (see unpublished Falbel et al. for detailed methods). The donor genome *Bacillus vallismortis* DV1-F-3 was selected due to its high collinearity and divergent average nucleotide identity of 90% against the recipient strain, *B. subtilis* PY79 (Fig. 1A). Despite the considerable divergence, the donor *Bacillus vallismortis* resulted in hundreds of transformants, comparable to yields obtained by an isogenic strain. Of hundreds of transformants obtained, this report describes the analysis of 11 sequenced transformant genomes (Table 1).

**TABLE 1:** Sequencing data generated in this work

Deposited sequence data	Transformation ID^*a*^	Genotype	Accession no.
*Bacillus vallismortis* DV1-F-3 genome	NA	NA	CP159908.1
PY79-5 (transformant 1)	1	WT	SRR29539908
PY79-1 (transformant 2)	1	WT	SRR29539907
PY79-4 (transformant 3)	1	WT	SRR29539905
2_BVPY792 (transformant 4)	1	WT	SRR29539904
PY79-6 (transformant 5)	1	WT	SRR29539903
1_BVPY791 (transformant 6)	1	WT	SRR29539902
3_BVPY793 (transformant 7)	1	WT	SRR29539901
2-7 (transformant 8)	2	WT	SRR29539900
PY79-3 (transformant 9)	1	WT	SRR29539899
2-3 (transformant 10)	2	WT	SRR29539898
BV-2-8 (transformant 11)	2	WT	SRR29539906
4_BVmutS4	1	*mutS*	SRR33623206
5_BVmutS5	1	*mutS*	SRR33623205
PY79-mutS-11	1	*mutS*	SRR33623204
PY79-mutS-12	1	*mutS*	SRR33623203
PY79-mutS-8	1	*mutS*	SRR33623202
PY79-mutS-9	1	*mutS*	SRR33623201
NR001-S6 (Syn transformant)	NA	NA	SRR29540657

^*a*^ Tansformation ID corresponds to transformants collected from the same day of transformation. All transformations were carried out under identical conditions.

During NT, donor DNA is recombined into the genome via double crossover events, where two recombination breakpoints flank the integrated donor DNA. We define the interval of identical bases between the outermost donor and recipient distinguishing SNPs as the crossover window, a region where crossover must have occurred (Fig. 1B).

The donor genome *B. vallismortis* DV1-F-3 contains well over 300,000 SNPs when aligned against *B. subtilis* PY79, with 99% of the SNP distances falling within 69 bp (mean 13 bp, median 6 bp). (Fig. 1C). While closely spaced SNPs increase the confidence of sites of crossing over, they present challenges for read-mapping algorithms that depend on the continuity of identical sequences. To maximize the potential for read pairs to map to a single reference genome and minimize read mapping artifacts, we mapped each transformant sample to a concatenated FASTA file containing both the donor and recipient chromosomes. Each base of the recipient genome was then assigned as originating from either the donor or recipient genome using a custom multinomial hidden Markov model (MHMM). The accuracy of these outputs was manually inspected and validated against a parallel aligned *de novo* assembly of each transformant genome. For details on the bioinformatic pipeline, MHMM specifications, and validation, see File S1 and Fig. S1.

Crossover windows on either side of the transferred donor DNA ranged from 1 to 2,591 bp, with a mean and median of 35 and 18 bp, respectively (Fig. 1D). Thus, the small degree of uncertainty surrounding sites of transformation allows for high-resolution analysis. A few outliers resulting from multicopy rRNA genes were omitted from subsequent analysis (Fig. 1D, red). The average crossover window length is approximately three times greater than the average SNP spacing distance (35 bp vs 13 bp), consistent with previous observations of higher identity surrounding recombination breakpoints (50, 53).

We next characterized the distribution of lengths for each of the 265 transferred segments in our data set. For this work, we define a transferred segment as the interval extending between the midpoints of the crossover windows on either side of the transferred segment. This can be conceptualized as artificially creating a recombination breakpoint at the middlemost base pair within each crossover window. The lengths of the donor transferred segments ranged from 7 to 36,229 bp (mean: 1,878 bp, median: 138 bp, excluding the ~2 kb *spcR* marker) (Fig. 1E). Over two-thirds of the transferred segments (68%) were under 1 kb in length, with 28% of the transferred segments being defined by a single SNP (Fig. 1F and Table 1). Importantly, the length distribution of the input donor DNA is markedly different from that of the transferred segments, as 97% of the input genomic DNA ranged from 10 to 180 kb with an average of 62,895 and a peak height of 51,602 bp (Fig. S2).

**Fig 2:**
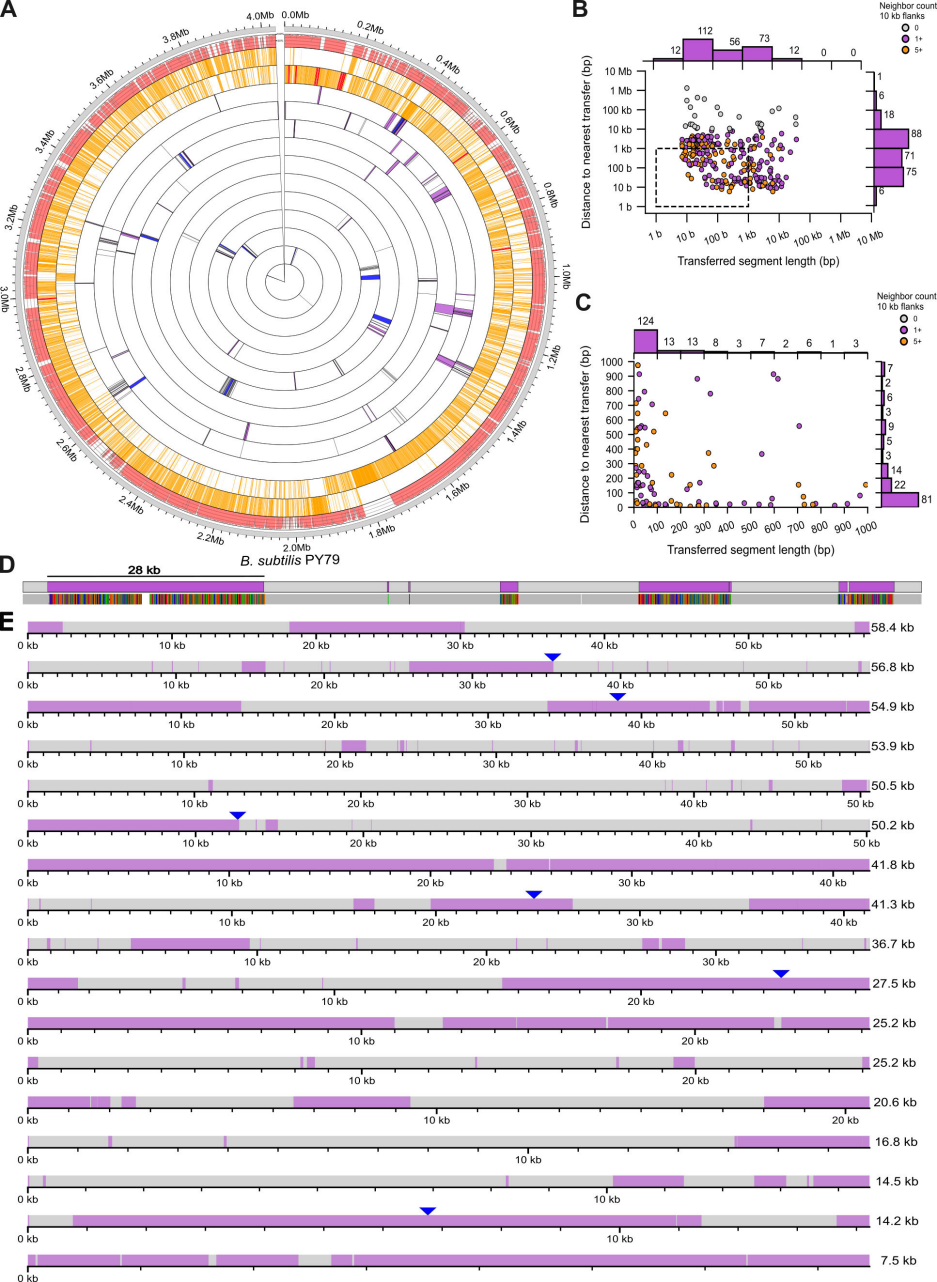
Mosaic patterns of natural transformation are frequent in *B. subtilis*. (**A**) Circos-style plot highlighting the site of homologous replacement in the recipient *B. subtilis* PY79 chromosome (blue: marker-present, purple: marker-absent) for each of the eleven transformants (innermost rings 1–11). Coding regions (orange) on the plus (ring 12) and minus (ring 13) strands are provided to designate *ori* and *ter*. Line plots corresponding to the percent identity within 300 bp windows are shown on the outermost track, with each gray gridline corresponding to 25% percent identity increments. (**B**) Marginal histogram depicting the lengths of transferred donor segments and their distance to the nearest transferred segment in the same transformant on a log scale. Each transferred segment is colored according to the number of neighboring transferred segments within 10 kb flanks on either side. The dashed box highlights distances and lengths that fall less than, or equal to 1 kb. (**C**) A subset of panel B for length values less than 1 kb. (**D**) An example of an individual transformant’s site of mosaic exchange where transformed and non-transformed regions lie in proximity on the recipient chromosome. The top row contains an example encoding of sites of transformation, where regions of purple rectangles denote sites of homologous replacement in the recipient. The bottom row is a visualization of the transformant contig in IGV, highlighting sites of dense SNPs (colored vertical lines) that are indicative of transferred donor alleles. (**E**) A representative selection of 17 highly mosaic clusters is displayed with donor transferred segments (purple) and their adjacent untransformed regions (gray). The *x*-axis displays relative coordinates to the start and end of the assigned clusters, with each minor tick on the *x*-axis corresponding to 1 kb in length. Positions of the selectable marker insertion site are displayed as a blue triangle where applicable.

Within an individual transformant, many transfer events cluster in proximity along the chromosome (Fig. 2A). Roughly half of the transferred segments lie within 1 kb of their closest neighbor (57%**;** Fig. 2B and C). Approximately 75% of the transferred segments have at least one neighboring transfer event within 10 kb (Fig. 2B, purple). Using the average length of the donor input library as a conservative guide, we next clustered sites of transformation within ~60 kb into groups to visualize and assess the confidence in their patterns of continuity (see File S1: Clustering and annotation of sites of transformation). Examining the frequency of donor alleles across sites of mosaic exchange, we found that each site of mosaic transfer is well-supported within the clustered groups, as their allele frequencies are consistent with the assignments of the MHMM (Fig. S3). An assembled transformant genome containing a representative mosaic cluster is shown in Fig. 2D. Several examples of highly mosaic clusters across multiple transformants and length scales are visualized in Fig. 2E. Importantly, using an independent transformation experiment transforming the marine cyanobacterium *Picosynechococcus* PCC 7002 with donor DNA harvested from a closely related *Picosynechococcus* PCC 11901, we show that mosaic patterns are not a result of *in vitro* preparation of the distributed marker library (Fig. S4). This experiment reinforces that mosaic outcomes are a cross-phyla phenomenon. Further analysis into potential effects of the selectable marker revealed that transferred segments containing a selectable marker were significantly longer in length and lower in percent identity than the marker-less transferred segments (Fig. S5A through D and Table S1). However, these lower percent identities are largely a product of insertions and deletions that can be attributed to the sampling of the genome itself across larger intervals (Fig. S5E).

### Short-length transfers deviate from proposed MEPS criteria

In the context of NT of *B. subtilis*, it has been proposed that a minimum of ~20 bp of perfect identity, also known as minimally efficient processing segments (MEPS), must be present on both ends of the invading DNA to initiate recombination (40). Similar length criteria have also been proposed in *Escherichia coli* (40, 54, 55). Functionally, when perfect identity is less than 100 bp, RecA-mediated strand exchange is orders of magnitude below optimal (54, 56) and readily reversible via ATP hydrolysis (57–59). Considering these proposed constraints to genetic recombination, we would predict short patches of recombination to be relatively rare in our data set. However, there were 124 transferred segments that were measured to be under 100 bp in length.

**Fig 3:**
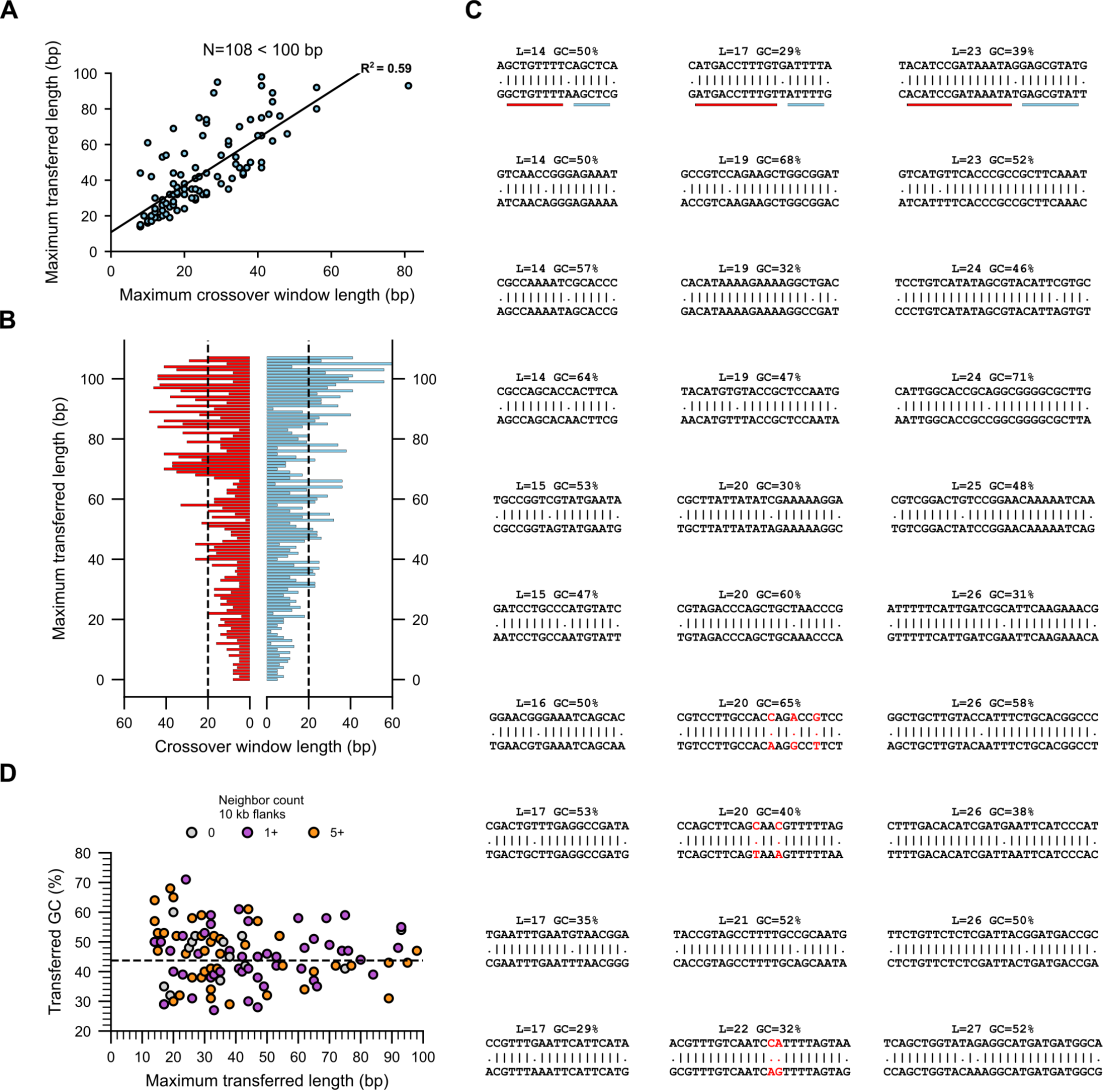
Short-length transfer events are inconsistent with the proposed MEPS for *B. subtilis*. (**A**) Linear regression fitting the longest crossover window of the transferred segment against the maximum total transferred length for short transfers less than 100 bp. (**B**) Horizontal bar plot visualizing the crossover window lengths for the 5′ end (red) and 3′ end (blue) for each of the transferred segments under 100 bp, sorted in descending order with respect to the maximum transferred segment lengths. Dashed vertical lines denote the proposed MEPS lengths of perfect identity with up to one mismatch. (**C**) Alignments of the 30 shortest transferred aligned against their orthologous recipient DNA. Each alignment begins and ends on the recipient defining SNP, with the internal nucleotides comprising the entirety of the donor transferred segment. Red and blue rectangles provide a visual of the crossover windows. The lengths and GC content are displayed for each alignment. Red highlighting denotes SNPs that exceed the minimum proposed MEPS criteria of one mismatch in ~21 bp. (**D**) Scatter plot of donor GC content and their respective lengths across each transferred segment under 100 bp in length (including the crossover windows), as well as the counts of their neighboring transferred segments in an individual transformant. The dashed horizontal line denotes the average GC content of the donor and recipient chromosomes.

To account for the fact that minor deviations in the length of the crossover windows could confound interpretations of which transfer events significantly deviate from expectations of efficient RecA-mediated strand exchange, we modified our analysis criteria to include the entire length of the crossover window (up until but not including the flanking recipient-defining SNP alleles). This accounts for the maximum possible MEPS. When including the entire crossover window, there were still 108 transfer events under 100 bp. These transferred segments were defined by one or more consecutive donor-specific alleles (1–11 SNP positions). For these short transfers, the longest of the two crossover windows for each transferred segment scaled linearly with the total length of the transferred segment (*R*^2^ = 0.59, *P* value = 2.90e–22), demonstrating that the observed short transfer events are largely a product of the crossover window lengths themselves (Fig. 3A). Plotting the crossover window lengths on each side of the integration events revealed a population of short transfer events where the entirety of the transferred segment was less than the proposed MEPS length (Fig. 3B).

Under the framework proposed by Majewski and Cohan (40), the MEPS for an invading end could be as short as 20 bp with up to one mismatch, provided that the GC content is greater than or equal to 52% (40). Using the MEPS framework, we examined the lengths of the 30 shortest transferred segments. Fourteen of the 30 segments were less than the proposed MEPS criteria, with 5 segments spanning 20–21 bp. Three segments exceeded one mismatch and several had GC content less than 50% (Fig. 3C). Furthermore, no significant correlation was found between the length of short transfers less than 100 bp and their GC content (Fig. 3D). Many of these short, transferred segments are proximate to other sites of transformation within the same transformant (Fig. 3D), suggesting that short transfers may be dependent on neighboring transformation events. These results raised the possibility that MEPS rules may be more permissive than previously described, or other mechanisms operating post-strand exchange may contribute to the short transfers. Thus, we hypothesized that these short transfer events, as well as mosaicism across multiple scales, could arise from MutS-mediated mismatch repair.

### Mosaic outcomes are not explained by mismatch repair

**Fig 4:**
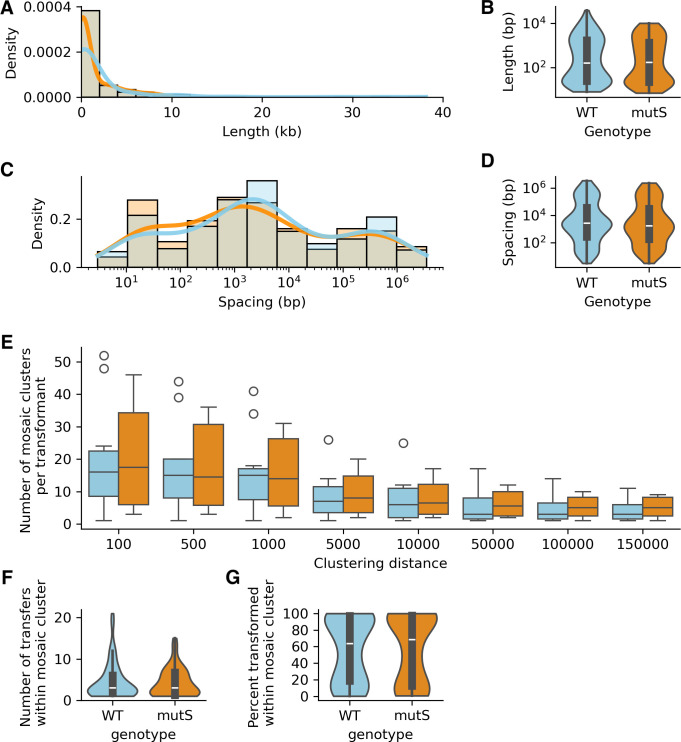
MutS does not explain mosaic outcomes of natural transformation. Summary statistics of WT (*N* = 11 transformants) and Δ*mutS* (*N* = 6 transformants). (**A and C**) Histograms and kernel density estimates enumerating the lengths of sites of transformation across the recipient genome (**A**) and the spacing between sites of transformation (**C**) for both WT (blue) and *mutS* (orange) backgrounds. (**B and D**) Violin plots of the lengths and spacing of sites of transformation, for the values shown in panels A and C. (**E**) Boxplots of the distributions of the number of clusters per transformant for a given merge distance parameter. (**F**) Violin plots of the number of transfers within a cluster group for both WT and *mutS*. (**G**) Violin plot for the percent of a cluster group’s total length that was transformed.

Upon sensing mismatches in the heteroduplex, mismatch repair systems can catalyze the excision of the invading donor DNA strand, and through long-patch repair, restore the original sequence using the recipient complement strand as a template (60). A potential consequence of this activity would be donor DNA segments being removed following strand exchange and replaced with recipient DNA. To test this model of mosaic genome generation, we compared transformant outcomes between a WT recipient and an isogenic recipient harboring a complete deletion of *mutS* (Δ*mutS*) (Fig. 4 and Table 1).

We first examined the distributions of the lengths of sites of transformation (Fig. 4A and B) and the spacing between sites of transformation along the recipient chromosome (Fig. 4C and D) between the WT and Δ*mutS* transformants. Neither the lengths of the sites of transformation (Mann-Whitney *U* statistic = 22,342.0, *P* value = 0.642) nor their spacing (Mann-Whitney *U* statistic = 24,527.5, *P* value = 0.434) differed significantly between the two recipient genotypes. Next, we asked whether the number of mosaic exchanges within a cluster was significantly different between WT and Δ*mutS* transformants. To assign clusters for this analysis, we used an unbiased approach, assessing how the number of clusters changes with respect to the distance parameter used to merge adjacent clusters (Fig. 4E). As the merging distance parameter increased in magnitude, the median and interquartile ranges of the number of clusters appear to stabilize between a merging distance of 10–50 kb for both the WT and Δ*mutS* genetic backgrounds. This is consistent with the observed peak length of 51,602 bp of our input donor DNA library (Fig. S2). For these reasons, we used the peak height corresponding to 51,602 bp as the distance to merge adjacent sites of transformation into a single cluster group for subsequent analysis. With respect to the degree of mosaic outcomes within cluster group assignments, the number of donor DNA segments within a cluster did not significantly differ between the two genotypes (Student’s *t*-test *P* value: 0.443) (Fig. 4F). Likewise, the percentage of the cluster group that corresponds to donor DNA did not significantly differ (Student’s *t*-test *P* value: 0.928) (Fig. 4G). These results suggest that *mutS* is not responsible for the observed lengths, spacing, or patterns of mosaicism across linked regions of transformation.

### Sites of mosaic exchange lack global and local genomic features

Next, we sought to identify if sequence-level features varied significantly between donor and recipient patches across 96 distinct cluster groups (WT: *N* = 58, *mutS: N* = 38) and whether these effects were dependent on the mismatch repair genotype. Overall, the distributions of percent identity, GC content, and the median RPKM gene expression (61) on a logarithmic scale (log_10_) appeared largely similar across both the genotype and genome of origin (Fig. S6). Given potential violations of independence among genomic intervals belonging to the same cluster group, we fit separate linear mixed-effect (LME) models for each local sequence feature of interest, using the cluster group itself as a random effect. Categorical variables denoting the genotype (WT vs Δ*mutS*), genome of origin (donor vs recipient), and bins of the lengths of the interval on a log-scale (0–10, 10–100, 100–1,000, and 1,000+ bp) were included as fixed effects for the LME model. The genotype was found to be non-significant in each of the models and was therefore omitted from the final fit models.

Overall, neither the genotype nor the genome of origin explained a significant proportion of variation of these local sequence features (Table 2 and Table S2 through S4). GC content was the exception, and it was found to interact with the length of the interval itself (Table 2). Performing pairwise comparisons across each length bin, the significant effect of the genome of origin was restricted to the 10–100 bp range, corresponding to a 4% higher GC content in short intervening recipient sequences when compared to short sites of transformation in the recipient genome (*P* value: 0.0001) (Table S5). More broadly, each of the fixed effects (length, genome of origin) explained less than 7% of the variance for each of the features of interest as evidenced by the estimated marginal *R*^2^ values generated from the fit LME model (Table 3). Likewise, when accounting for the random effect of the cluster group (conditional *R*^2^), there was an approximately equal or greater contribution from the cluster group itself, suggesting that factors beyond the identity of the interval, the genotype, or the cluster group explain the observed variations of the aggregate local sequence features (Table 3). The intraclass-correlation coefficients (ICCs) for each of the LME models corresponded to a value less than 10%, suggesting that the variation within a cluster group is greater than the variation between cluster groups (Table 3). These results are further supported by several transformants displaying distinct mosaic outcomes despite overlapping the same region in the recipient, suggesting that percent identity, GC content, and gene expression are not strong determinants of outcomes of mosaic exchange (Fig. S7). Thus, global summaries of percent identity, GC content, and median gene expression do not significantly differ between sites of transformation and their intervening recipient-derived DNA.

**TABLE 2:** *P* values for the linear mixed-effect models for percent identity, GC content, and log_10_ median RPKM using a type III analysis of variance via Satterthwaite’s method^*a*^

	Percent identity	GC content	Log_10_ median RPKM
Genome	0.899	0.03584*	0.595
Length	7.64e−10***	0.17331	0.996
Genome:Length	0.233	0.01040*	0.800

^*a*^ *P < 0.05, **P < 0.01, ***P < 0.001. Bolded values delineate significant *P*-values (*P* < 0.05).

**TABLE 3:** Estimates of *R*^2^ and the intraclass correlation coefficients for the linear mixed-effect models for percent identity, GC content, and log_10_ median RPKM

	Percent identity	GC content	Log_10_ median RPKM
*R*^2^ marginal	6.88%	3.76%	0.13%
*R*^2^ conditional	12.09%	9.71%	26.39%
Adjusted ICC	5.6%	6.2%	2.6%
Unadjusted ICC	5.2%	5.9%	2.6%

**Fig 5:**
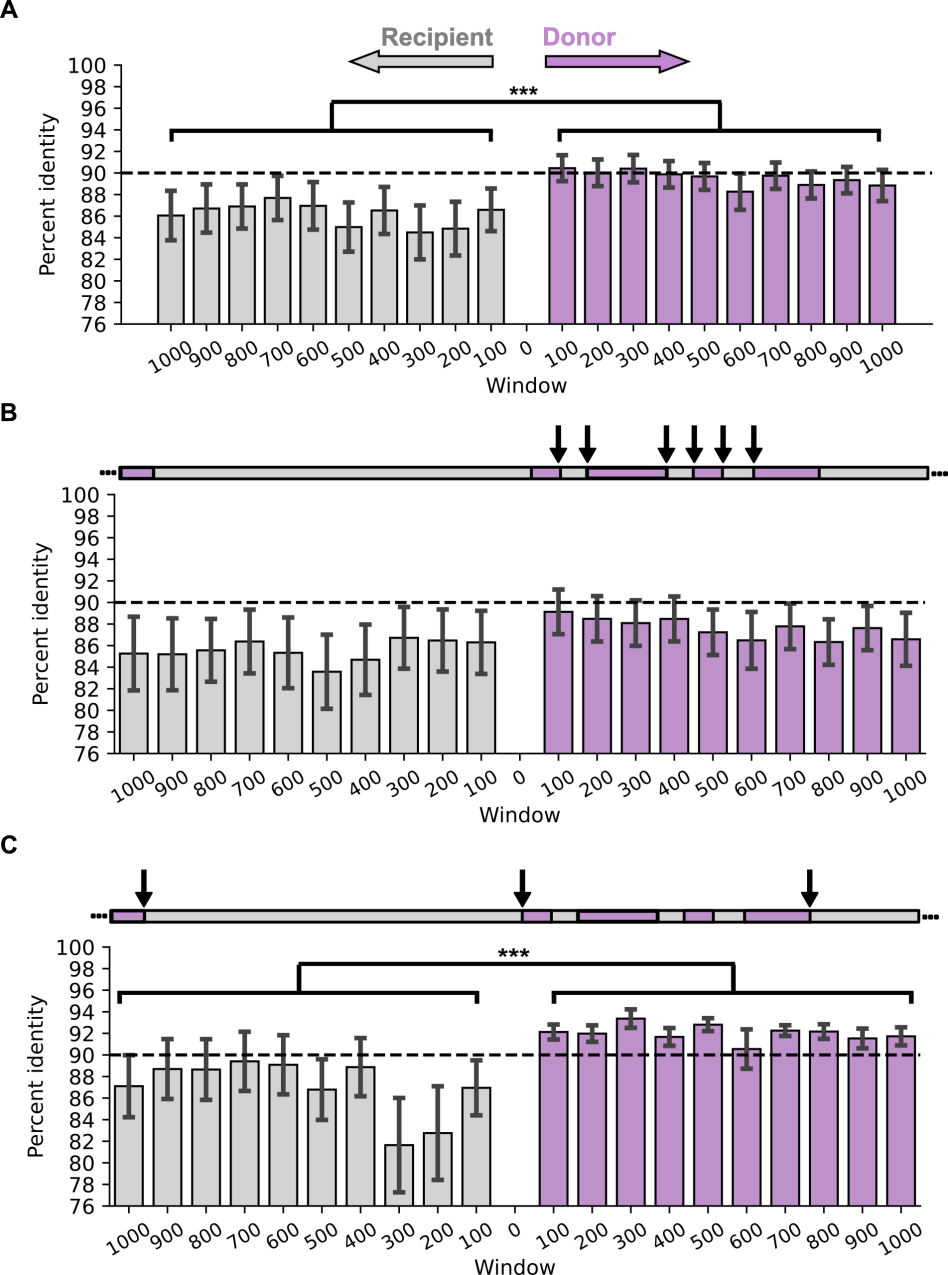
Mosaic sites of transformation lack distinct signatures of percent identity surrounding recombination breakpoints. (**A-C**) Bar plots displaying the average percent identity and their standard error of the mean for 100 bp windows flanking the recombination breakpoints (donor side purple, recipient side gray). Separate analyses were performed to distinguish between mosaic versus non-mosaic breakpoints with significant differences noted (***P < 0.001). (**A**) Recombination breakpoints for all sites of transformation and intervening sequences exceeding 1,000 bp (*N* = 164). (**B**) A subset of panel A corresponding to recombination breakpoints with adjacent intervening recipient sequences between 1,000 bp and the peak input DNA length (1,000–51,602 bp; *N* = 92). (**C**) A subset of panel A corresponding to recombination breakpoints with adjacent intervening recipient sequences that exceed the peak input DNA length (>51,602 bp; *N* = 72).

We next considered whether local patterns of sequence divergence proximal to sites of transformation may act as roadblocks that halt strand exchange. To probe for signatures of high divergence surrounding sites of transformation, we calculated the percent identity within 100 bp genomic windows totaling 1 kb (size: 100, step: 100, and windows: 10) encapsulating the recipient-derived DNA and the donor-derived DNA surrounding each midpoint of each crossover window (breakpoint). To prevent flanking windows from overlapping both recipient and donor-derived DNA, or the same region several times, we omitted breakpoints where the length of the site of transformation, or the corresponding intervening recipient sequence, was less than 1 kb in length. Thus, each window is guaranteed to cover a given region of the recipient in an individual transformant once and only once. The average BLAST-like percent identity for each window according to the genome of origin is visualized (Fig. 5A). A two-way ANOVA (type III sum of squares) was performed to compare the percent identity for each 100 bp window, with the window number and the genome of origin as main effects including the potential for interaction (Table S6 through S8). The average percent identity of the donor-derived windows (89.55%) was significantly greater than those of the recipient-derived DNA (86.17%). No significant effect of the position of the window relative to the breakpoint was identified in either genome of origin (Table 4).

**TABLE 4:** *P* values derived from the two-way ANOVA analysis of percent identity within sliding windows surrounding recombination breakpoints across mosaic and non-mosaic subgroupings^*a*^

	Mixed group (1,000–51,602+ bp)	Mosaic group (1,000–51,602 bp)	Non-mosaic group (>51,602 bp)
Genome	3.70e−05***	0.086	1.29e−06***
Window	0.998	0.997	0.860
Genome:Window	0.962	1.0	0.458

^*a*^ *P < 0.05, **P < 0.01, ***P < 0.001. Bolded values delineate significant *P*-values (*P* < 0.05).

To better understand this relationship in the context of mosaic exchange, we designated each recombination breakpoint as belonging to a mosaic grouping if their adjacent intervening recipient length fell between 1,000 bp and the peak length of our input donor DNA (51,602 bp) (Fig. 5B), or to a non-mosaic grouping if their adjacent intervening recipient DNA length exceeded 51,602 bp, as these would be less likely to arise from a continuous strand of input DNA given our input size range (Fig. 5C).

For recombination breakpoints classified as mosaic, neither the genome of origin nor the window position was found to be significant (Table 4). In contrast, within non-mosaic sites of transformation, the average percent identity of the donor-derived windows (92%) was significantly higher than the recipient-derived DNA (86%) (*P* value: 1.29e−06). We rationalized that the omission of the shared 1,000 bp intervening recipient sequences would not confound our downstream analysis since their average percent identity (91.72%) slightly exceeded the average nucleotide identity between the two genomes (90%) (Fig. S8). Importantly, these results were consistent when the analysis was repeated when allowing for overlapping windows, requiring a minimum of 200 bp for sites of transformation and their adjacent intervening sequences (Table S9 through S11). Overall, these results demonstrate that sites of mosaic exchange lack significant differences with respect to the fraction of identical bases, GC content, and median gene expression.

## DISCUSSION

Starting from the pre-sequencing era, researchers have observed discontinuities in outcomes of natural transformation (23, 24, 62). More recently, discontinuous exchange has been identified from whole-genome analysis of transformants from *B. subtilis* (14, 22, 52), *Streptococcus pneumoniae* (47, 48), *Haemophilus influenzae* (50), and *Helicobacter pylori* (53). This work leverages a new computational and analytical framework to resolve individual parental alleles across sites of transformation. Through this approach, we observed a breadth of lengths and continuities resulting from single rounds of natural transformation. Across several transformant genomes, we report clusters of highly mosaic sites of transformation that were independent of MutS-mediated mismatch repair. These results further support mosaic outcomes of transformation as a cross-phylum phenomenon, raising the potential for universal molecular features that drive mosaicism and allelic diversity.

We identified mosaic outcomes across multiple scales, in particular numerous short transfer events less than 100 bp in length. Short-length transfers have been reported in both single-round transformations in *H. pylori* (53), as well as transformants derived from iterative rounds of transformation in *B. subtilis* (22). To our knowledge, this is the first work in *B. subtilis* to report sequence identity and GC content features surrounding short-length transfers in *B. subtilis* and to interrogate potential mechanisms. Many of these short transfers were inconsistent with the proposed minimum requirements of a MEPS to initiate homologous recombination (40).

Therefore, we tested a model where intervening recipient patches could be the result of MutS-mediated repair processes on the heteroduplex. Patterns of mosaic exchange did not significantly differ between transformants derived from WT and Δ*mutS* recipient strains. These results do not exclude or refute the potential for mismatch repair to contribute to certain patterns of mosaic exchange in broader contexts and taxonomy. However, they do point to other mechanisms that generate discontinuity.

Given the lack of evidence for mismatch repair contributing to observed patterns of mosaic outcomes, we next examined whether genomic features may distinguish between donor and recipient in sites of mosaic exchange. Linear mixed-effect models using the length of the interval and its genome of origin explained less than 7% of the total variation. This suggests that global percent identity, GC content, and gene expression are inadequate for predicting mosaic exchange. Conversely, non-mosaic sites of transformation harbor higher percent identity within the donor-derived regions surrounding recombination endpoints compared to their surrounding recipient-derived regions. This observation is consistent with previous reports of higher identity at the recombination breakpoints surrounding sites of transformation (50, 53). Altogether, we interpret these results to suggest that sites of mosaic transformation do not result from independent strand exchange events, but rather, from modifications to the heteroduplex.

In theory, several non-mutually exclusive models could explain the patterns of discontinuous transformation. During DNA uptake, extracellular and intracellular nucleases could cleave the incoming donor. After translocation, the homology search is regulated by a myriad of recombination and mismatch repair proteins that modulate RecA-mediated strand exchange (41, 63). Through their interactions, one or more protein regulators could act to alter the continuity in the transforming DNA. Following the formation of a stable heteroduplex, the invading donor DNA strand is a potential substrate for cleavage, resection, and repair by DNA repair enzymes and restriction modification systems (64–66). Furthermore, short-length transfers could arise through homology-dependent illegitimate recombination, where sites of transformation can occur indirectly via RecA-mediated strand exchange on nearby patches of homology (67–71). These mechanisms may independently or synergistically result in mosaic outcomes. The molecular machinery and features that drive these mosaic outcomes across broad stretches of transformed DNA remain to be elucidated. In particular, to adequately model such spatially complex causative factors, analysis of hundreds of transformants is likely warranted.

More generally, our results indicate that the breadth of exchange that occurs in complex communities via natural transformation has been underestimated. Probabilistic models of natural transformation, such as the MHMM developed in this work, will ultimately improve both the genotypic analysis of gene exchange outcomes and their phenotypic interpretation.

## MATERIALS AND METHODS

### Bacterial strains and general growth conditions

All strains used in this study are listed in Table 5. Construction of distributed marker libraries and natural transformation of *B. subtilis* and *Picosynechococcus* were carried out as described in Falbel et al. (unpublished). *B. subtilis* transformant colonies were harvested directly from selective agar media (LB + spectinomycin 100 µg/mL), and resuspended in 100 µL of 20 mM Tris, 50 mM EDTA, 100 mM NaCl lysis buffer for genomic DNA isolation. Cultures of *Picosynechococcus* were grown in six-well tissue culture plates in 5.5 mL of AD7 media under continuous LED illumination (~70 µmol/m^2^·s PPFD) at ambient CO_2_ with shaking (130 rpm) at 30°C until the cells had grown to a density high enough to obtain a cell pellet of about 50 mg for genomic DNA isolation.

**TABLE 5:** *Bacillus* and *Picosynechococcus* strains used in this study

Strain	Stock center ID	Genome accession no.
*Bacillus subtilis* subsp. *subtilis* PY79	1A747	CP006881.1
*Bacillus vallismortis* DV1-F-3	28A4	CP159908.1
*Picosynechococcus* sp. PCC 7002	PCC 7002	GCF_000019485.1
*Picosynechococcus* sp. PCC 11901	PCC 11901	GCF_005577135.1

The Δ*mutS* recipient strain was generated from a full *mutS* deletion obtained from the Koo et al. knockout collection (72), followed by two rounds of backcrossing into PY79 to obtain a Δ*mutS* strain that is largely isogenic to WT PY79. Full *mutS* gene deletions were confirmed via manual inspection of a lack of coverage within the *mutS* coding sequence after mapping the reads of the transformant to the concatenated reference FASTA file for each of the Δ*mutS* transformants.

### Transformant genomic DNA extraction, purification, and sequencing

Sequencing of *B. subtilis* transformants was performed using a standard phenol/chloroform extraction (28), and then purified with the Zymo Quick-DNA HMW Mag Bead kit (D6060) DNA purification protocol according to the manufacturer’s instructions. Library preparation of transformant genomes via tagmentation was performed using the Illumina Nextera XT library prep kit and compatible 10-nucleotide UDI primers (Integrated DNA Technologies). Samples were size-selected and purified on JetSeq Clean magnetic beads and subsequently sequenced on an Illumina NovaSeq at the University of Wisconsin-Madison Biotechnology Center, at a 2 × 150 run aiming for 2 million paired-end reads per genome.

*Picosynechococcus* transformant cell pellets were sent to SeqCenter (Pittsburgh, PA, USA), who isolated genomic DNA from cells using the ZymoBIOMICS DNA miniprep kit with the modification for elution incubating in 75 µL water for 5 min (rather than 1 min) before centrifugation. Illumina sequencing libraries were prepared using the tagmentation-based and PCR-based Illumina DNA Prep kit and custom IDT 10bp unique dual indices (UDIs) with a target insert size of 320 bp. Illumina sequencing was performed on an Illumina NovaSeq 6000 sequencer in one or more multiplexed shared-flow-cell runs, producing 2 × 151 bp paired-end reads. Demultiplexing, quality control, and adapter trimming were performed with bcl-convert1 (v4.1.5). Accessions for each of the sequenced transformants are provided in Table 1.

HMW DNA extraction and sequencing for the *B. vallismortis* DV1-F-3 genome are described in File S1.

## Data Availability

Raw sequencing data for each of the transformant genomes are deposited in the SRA database and referenced in Table 2. All code used to perform the MHMM decoding, analysis of recombined segments, and statistical testing is deposited under doi:10.6084/m9.figshare.26307595. Jupyter notebooks are provided for full reproducibility of the generation of figures and analyses. Transposon vector C is available upon request.

## SUPPLEMENTAL MATERIAL

File S1 mbio.00444-26-s0001.pdf Extended bioinformatic methods, Table S1, and Figures S1-S7.

File S2 mbio.00444-26-s0002.xlsx Locations of the 9-bp duplication (insertion site) of the spcR marker across each of the eleven WT transformants.

File S3 mbio.00444-26-s0003.txt Transferred segments defining the start and end by the innermost donor SNP alleles for WT samples.

File S4 mbio.00444-26-s0004.txt Transferred segments defining the start and end by the midpoint of the crossover window for WT samples.

File S5 mbio.00444-26-s0005.txt Transferred segments defining the start and end by the outermost base of the crossover window for WT samples.

File S6 mbio.00444-26-s0006.txt WT and MutS data used in the analysis.

Supplemental Material Descriptions mbio.00444-26-s0007.pdf Descriptions for each supplemental file.

ASM does not own the copyrights to Supplemental Material that may be linked to, or accessed through, an article. The authors have granted ASM a non-exclusive, world-wide license to publish the Supplemental Material files. Please contact the corresponding author directly for reuse.
