# Supplementary material for: High-resolution genomic analysis reveals abundant mosaic outcomes of bacterial natural transformation independent of MutS-mediated mismatch repair
Source: mBio. 2026 Jun 15;17(7):e00444-26. doi: 10.1128/mbio.00444-26 (PMC13344027; doi:10.1128/mbio.00444-26)
Supplement: File S1 — Extended bioinformatic methods, Table S1, and Figures S1-S7. [file mbio.00444-26-s0001.pdf]

Supplemental File S1

High-resolution Genomic Analysis Reveals Abundant Mosaic Outcomes of Bacterial Natural Transformation Independent of MutS-mediated Mismatch Repair

Jonathan M. Lombardino,[a,b] Tanya G. Falbel,[a] Colin N. Dewey,[c] Briana M. Burton[a,#]

[a]Department of Bacteriology, University of Wisconsin - Madison, Madison, WI
[b]Microbiology Doctoral Training Program, University of Wisconsin - Madison, Madison, WI
[c]Department of Biostatistics and Medical Informatics, University of Wisconsin - Madison, Madison, WI
[#]Address correspondence to Briana M. Burton, briana.burton@wisc.edu.

Table of Contents

Supplemental Methods
Supplemental Results
Supplemental References
Supplemental Table S1-S11
Supplemental Figures S1-S8

Supplemental Methods

HMW DNA quality assessment

Quantification of the extracted Bacillus DNA was measured using the Qubit™ dsDNA High Sensitivity kit (ThermoFisher Scientific). Samples were diluted before running on the Agilent Fragment Analyzer using the HS Large Fragment 50Kb kit to assess DNA size and quality at the UW Biotechnology center.

Genomic DNA extraction for PacBio sequencing

High molecular weight target donor genomic DNA was isolated from *Bacillus subtilis* PY79 or *Bacillus vallismortis* (CP159908) using phenol/chloroform-based extraction, followed by ethanol precipitation, RNAse A digestion, an additional chloroform extraction and re-precipitation. Please note that in our hands, spin column kit-purified genomic DNAs contained contaminants (likely residual proteinase K) that inhibited the transposition reaction. Phenol/chloroform extraction was found to be superior in this method. We did not evaluate whether including extra rinse steps from proteinase K based preparations would overcome this inhibition.

PacBio sequencing and reference genome assembly

The reference sequence for *Bacillus vallismortis* DV1-F-3 was generated using long-read PacBio HiFi Circular Consensus Sequencing (CCS) libraries generated according to PN 101-696-100 version 01 (Pacific Biosciences). Input genomic DNA was measured on a NanoDrop™ 1000. Samples were diluted before running on Agilent Fragment Analyzer using the HS Large Fragment 50Kb kit to assess DNA size and quality at the UW Biotechnology center. First, an initial *de novo* assembly was performed using HGAP, producing a single circular chromosome and a small plasmid of length 8,533 bp.

In parallel, an Illumina 2x150 sequencing library was generated to perform error correction and polishing of the resulting *B. vallismortis* DV1-F-3 genome assembly. Reads were aligned to the draft genome assembly using the Geneious read mapping software to identify potential variants. Each variant was manually inspected using the Geneious software, replacing

single nucleotide variants and short indels if the allele frequency of the alternate allele was over 90% and the variant allele depth was greater than 10. Additionally, a 23 kb duplicated region was removed due to a lack of supporting coverage in the Illumina dataset. Another iteration of read mapping was performed using the same reads, to confirm support for each of the altered variant alleles. This genome sequence was deposited under bioproject PRJNA1130923 and accession CP159908.1 (chromosome), CP159909.1 (plasmid).

Due to the presence of a pre-existing genome sequence for *B. subtilis* PY79, PacBio sequencing and *de novo* assembly was unnecessary. Instead, an Illumina 2x150 sequencing library of our lab's *B. subtilis* PY79 was generated and mapped to the NCBI reference chromosome (CP006881.1). Two single nucleotide polymorphisms (SNPs) were identified at positions 1683303 (C>T), and 3528887 (T>C). The resulting chromosome was saved to a file titled PY79.fasta which was used as the recipient reference sequence.

Whole genome alignment and average nucleotide identity calculations:

Whole genome alignment (WGA) was carried out using progressiveMauve v2.4.0_snapshot_2015_02_13 with the command "progressiveMauve {PY79.fasta} {BV.fasta} –seed-weight=24" to generate an output XMFA file. An optimal seed weight of 24 was chosen after manual inspection of the resulting locally collinear blocks (LCBs). These LCBs were largely consistent with other *Bacillus* genome alignments to *B. subtilis* PY79 (data not shown). With the harbinger_wga.py script, a liftover table was constructed for every 1-based position and their aligned base in the recipient chromosome, along with every orthologous base and their position in the donor *B. vallismortis* DV1-F-3 chromosome. Additionally, along with the liftover table, a cigar alignment (=: Match; X: mismatch; I: insertion in the donor; D: insertion in the recipient) for each LCB was included in PAF (Pairwise Alignment Format) and SAM (Sequence Alignment Map) formats. Because a LCB generated by progressiveMauve can include regions of long non-homologous material, more traditional, reciprocal BLAST-based approaches were better suited to capture the "average" identity of homologous regions. Average nucleotide identities between each of the donors and their respective recipient were performed using the Kosta lab's average nucleotide webserver (http://enve-omics.ce.gatech.edu/ani) using the default settings (1).

<u>Transformant *de novo* assembly, read mapping and variant calling:</u>

Adapter removal/trimming for the raw sequences was performed using bbduk.sh v39.01 (mink=12 ktrim=r k=21 hdist=1 ref=resources/adapters.fa). Following adapter trimming, *de novo* assemblies of each trimmed fastq pair were carried out using spades v3.15.1 using the –isolate preset. To maximize the probability that any divergent read or contig could map to at least one parental genome sequence, a concatenated reference fasta containing both the *B. vallismortis* DV1-F-3 and *B. subtilis* PY79 genome was created. The *de novo* contigs were mapped to this concatenated reference sequence using minimap2 v2.26-r1175 with the command: "minimap2 -x asm20 -a -L -c --cs --MD --eqx -Y {genomes/all_genomes.fasta} {sample contigs.fa}". In parallel, the adapter-trimmed short reads were mapped to the same concatenated reference files using minimap2 with the command "minimap2 -ax sr --eqx -t 20 {genomes/all_genomes.fasta}. Next, variants were encoded in vcf format using bcftools v1.17 using the command "bcftools mpileup -I -A -a AD,QS -d 50 –threads 20 -f genomes/all_genomes.fasta {sample.bam} | bcftools call -c --keep-alts --ploidy 1 --threads 20 | bcftools filter -e DP<3 -o {sample.vcf.gz}." These resulting vcf files were used as input to the harbinger_model.py script.

For the *Picosynechococcus* transformant derived from the donor strain NR001 **(**FEK30_03020::*spcR*), adapter trimming and *de novo* assembly was carried out using the same approach as the *B. subtilis* transformants outlined above. The resulting contigs were then mapped to the recipient genome of *Picosynechococcus* sp. PCC 7002, using the minimap2 software with the following command: "minimap2 -a --eqx -t 20 -x asm20 PCC7002_GCF_000019485.1_ASM1948v1_genomic.fna {sample}.fa | samtools view -bh | samtools sort -o {sample}.to_7002.sorted.bam". The resulting output bam file was visualized in IGV and reported in **Figure S4.**

<u>Allele counts liftover and Multinomial Hidden Markov Model decoding.</u>

A 4033459x5 matrix of ones was initialized with every row corresponding to a position in the recipient genome, and every column representing each of the possible alignment characters (A,C,G,T,-). Each transformant VCF was separately parsed using the harbinger_model.py script, with each of the recipient VCF entry's allelic counts being transferred to their corresponding row of the chromosome matrix. For each donor chromosome VCF entry, sites of coverage were "lifted over" to their orthologous bases, if any were present. Since all VCF file positions and allele entries correspond to the forward strand, any donor VCF entries that spanned a negative strand alignment according to the WGA had their allelic counts reverse complemented. Finally, all positions in the matrix were used as input to the Multinomial Hidden Markov Model (MHMM).

An MHMM was constructed to predict donor and recipient genome states based on the observed counts of each possible nucleotide across every position of the recipient genome. Two parameters were created to govern the state transitions between the donor and recipient genome (**Figure S MHMM model A**). The parameter α specifies the probability of not initiating a recombination event in the next position of the recipient genome, which ultimately governs the lengths between recombined segments (Figure **S MHMM model B**). Conversely, the parameter β governs the length of recombined segments, encoding the probability that the next base will continue to stay in a recombined segment in the donor state (**Figure S MHMM model B**). The emission probability function of the HMM follows a multinomial distribution and is depicted below in Equation 1.

Equation 1

$$e_{ki}(x_i) = \prod_j \theta_{R_{ik},j}^{x_{ij}}$$

The factorial term of the multinomial probability function was omitted for efficient calculations, as it is a constant that depends only on the data and doesn't influence the relative probability of either state. Let x denote a vector of observed base counts for every jth base (A,C,G,T,-) across each of the ith positions in the recipient genome, and let k represent the index

of the donor and recipient genome states (recipient: 0, donor: 1). R_{ik} corresponds to the base at position i, in the kth genome state, and θ represents the probability of observing base j in a read when the true genome base is R_{ik}. A simple error matrix for θ was constructed using a single parameter that governs the probability of sequencing error, ϵ. When an observed base and the reference allele are equal, the probability that the observed base did not result from sequencing error can be defined as θ = 1 - ϵ. If the observed base is found to differ from the reference allele, the probability that the base did not result from a sequencing error is defined as $\theta = \frac{\epsilon}{n-1}$, where n is the number of potential allele characters (A,C,G,T,-) such that each row of the error matrix sums to a probability of 1. We found that α=0.99, β =0.99, ϵ =0.01, were optimal parameters for the MHMM that agreed with the observed outputs of the mapped *de novo* assembled contigs and short paired-end reads.

Decoding of the parental origin of each position in the genome was carried out through an implementation of the Viterbi algorithm. Because the recipient genome contained positions with gaps in the donor, the count of gap characters couldn't be accurately modeled from a multinomial emission probability with liftover counts. At these positions, the multinomial probability of the donor was set to be equal to the recipient's. We reasoned that the Viterbi algorithm would generate useful outputs so long as there was reliable coverage of nearby SNP positions. Although the Viterbi path was calculated using data from all the recipient genome positions, we assigned recombined segments by grouping consecutive donor states only at the positions where the recipient genome differed from the donor (i.e. orthologous SNPs). We found that by grouping at SNP positions, we could minimally define the unit of transfer as the innermost SNP positions that allow for addition of the flanking crossover windows for certain analyses. Additionally, spurious transitions surrounding hard-to-assess gapped characters were avoided through this conservative decoding. Thus, for each recombined segment, the start and end positions were always SNP positions that were decoded as the donor allele (**Figure S1 MHMM model 1C**). Ultimately, this computational workflow identified discrete intervals of transferred bases, defining a unit of transfer as segments of continuous donor SNP alleles that are uninterrupted by a recipient-specific allele. Under this model, the minimum length of a transferred unit could comprise a single donor SNP allele. Likewise, a single well-supported recipient SNP allele can break a stretch of donor DNA sequence

into two separate donor DNA segments. To characterize the mosaic nature of these events, it was of utmost importance to have a minimal classification of continuous transfer.

The intervals derived from the MHMM decoding were output in the form of a PAF file for each individual transformant sample. Every output was manually validated by comparing these intervals against both the mapped paired-end reads, as well as the *de novo* assembled contigs in IGV. Of note, one of the samples had a transformation event that spanned the origin, and was manually split into two records, with a new record starting on the first SNP position in the WGA. While this implementation was satisfactory for our experimental conditions, we caution readers in the use of this version of HARBINGER outside of these exact strain pairings and library quality. See TheBurtonLab/HARBINGER GitHub repository for the up-to-date release version.

Quantifying lengths and proximity of transferred segments:

For most of the analyses performed in this work, we defined the length of the transferred donor segments as the distance between each midpoint of the crossover windows (rounding up if the midpoint position fell between two base pairs). In other words, we defined the recombination breakpoint as the middlemost base pair between the crossover window boundaries.
However, we also employed a length measurement scheme where the entirety of the crossover windows was included in the calculation for short, transferred segments under 100 bp. For each transformant, the proximity was calculated by sorting the intervals of the donor transferred segment, or recipient sites of replacement, in ascending order. For each interval, the intervening distance to the next and proceeding interval was calculated, defining the minimum of these two intervening distances as the “closest” or “nearest”. Additionally, the number of neighboring segments or sites of homologous recombination was calculated by creating a 10 kb window on both sides of the interval, counting the number of other intervals that overlapped with queried one.

For analysis comparing transferred segments based on the presence or absence of the marker, the length of the inserted marker and the resulting 9 bp duplication of transposition (total of 2,002 bp) was added to the length of only the donor transferred genomic segments that contained the marker resistance cassette.

Marker insertion site identification:

The presence of markers in the WT samples was manually identified according to Falbel et al. (unpublished), by confirming the presence of mosaic ends in the forward and reverse orientation, separated by a hallmark 9 bp duplication that is generated during transposition. The sites of *spcR* insertion by transposition in each of the WT transformants were recorded as genomic coordinates of the donor genome *B. vallismortis* DV1-F-3 and are provided in **Supplemental File 2**. These marker insertion sites were then confirmed to be entirely encompassed in the identified donor transferred segments and added as a binary label to the recombined segments.

Percent identity calculations:

Orthologous alignments between the donor and recipient chromosomes were precomputed and stored as a PAF and SAM file and were represented as a cigar string. Custom python code was written to index these cigar string operations by the recipient or donor base pair positions. For each transferred segment, the donor coordinates were used to slice the WGA cigar alignments into an appropriate subset that captured only the alignment columns that spanned the transferred donor DNA sequence. To calculate percent identity, two separate methods were used. A BLAST-like identity was performed by taking the sum of the matching characters and dividing them by the number of matches, mismatches and indels.

$$blast\ identity\ = \frac{|matches|}{|matches|\ +\ |mismatches|\ +\ |Insertions|\ +\ |Deletions|}$$

Additionally, a second identity calculation was performed using a gap-compressed method. Conceptually, gap-compressed percent identities treat consecutive indels as a single gap open in the calculation which "compresses" the alignment columns where there are runs of gapped sequences.

$$gap - compressed\ identity\ = \frac{|matches|}{|matches|\ +\ |mismatches|\ +\ |gap\ opens|}$$

$$Gapless - Identity = \frac{|matches|}{|matches|+|mismatches|}$$

Genome background sampling:

Random sampling of the WGA was performed by iterating through the length distribution of the transferred segments that harbor selective markers (*spcR*+ inclusive). LCBs that were sufficiently long to include the entirety of the sampled length were included, selecting one at random. Then, a random donor coordinate interval matching the sampled length was created, slicing the cigar to create a subset of the alignment.

Visualizing gene annotations, GC content, sequence variants, and competence RNA-Seq:

Refseq gene feature fasta (GFF) files containing annotations for the *B. subtilis* PY79 genome assembly (GCF_000497485.1) and the reference sequence *B. subtilis* 168 (GCF_000009045.1) genomes were retrieved from NCBI. For analyses incorporating gene expression, a larger annotated dataset was curated using external annotations obtained from *B. subtilis* 168 and is summarized as follows. To visualize gene expression data originating from the competent cell-state of *B. subtilis* 168, externally generated RNA-seq data was obtained from Boonstra et al. 2020 (2). To generate a single value to represent expression levels, the average across two biological replicates per timepoint were generated. Then, a second average was taken across each of these averaged timepoints. Gene expression annotations were then integrated in the *B. subtilis* 168 GFF data in bed file format to map the coordinates of each annotation between the *B. subtilis* PY79 and 168 strains using the paftools.js liftover subcommand (unimap -x asm20 GCF_000497485.1_ASM49748v1_genomic.fna GCF_000009045.1_ASM904v1_genomic.fna -c --cs | paftools.js liftover -q 0 -l 0 -d 2 - BSUB168_to_liftover.bed > PY79_liftover.bed).

To represent GC content and sequence variant density for feature pileups, non-overlapping sliding window intervals for the *B. subtilis* PY79 genome were generated using bedtools makewindows (stepsize=100, windowsize=100). These windows were then used as input to obtain the GC content (bedtools nuc) and count the number of variants that overlapped within each window (bedtools intersect).

Clustering and annotation of sites of transformation.

For each transformant, an initial conservative approach was used to cluster mosaic sites of transformation against the average input gDNA length. These cluster groups were created using the raw outputs of each of the PAF files and were sorted according to the recipient coordinates. A grouping of these conservatively clustered transferred segments was allowed to grow in number so long as the total length in the donor or recipient did not exceed 60 kb, the intervening distance between one segment and the next in the queue did not exceed 30 kb, the next segment in the queue did not correspond to a genome rearrangement, and the strands of the next donor segment in the queue was the same as the previous. After assigning on a transformant-by-transformant basis, all cluster group assignments were then appended into a single list for all transformants. Next, we scored each cluster group for interesting patterns of mosaicism through a heuristic score that raised the total transferred donor base pairs in the cluster group to the number of independent events. This heuristic proved useful for visualizing long tracts of transfer that were frequently in proximity.

For analysis of the features of within and between sites of mosaic exchange, a more lenient clustering approach was conducted. For every transformant, cluster groups of sites of transformation across the recipient genome were created using bedtools v2.30.0 via the following pipeline: bedtools sort -I {input file} | bedtools spacing -i stdin | bedtools cluster -i stdin -d {distance} > {output file}. Custom python code was used to generate every interval intervening between sites of mosaic transformation, labeling each interval of the recipient as being transformed, belonging within a given cluster (intralinkage) and spanning between mosaic clusters (interlinkage). Using an id in the bed format, each recipient interval could be isolated according to the mosaic cluster it belongs to, if any.

Next, bedtools was used to retrieve features associated with each genomic interval. GC content for each interval was obtained via bedtools nuc, and summary statistics associated with the RPKM of overlapping genes was obtained via bedtools map -c 4 -o mean,stdev,median,min,max,sum,count. For percent identity, the progressiveMauve xmfa file was converted into a featurized bed file (BV_vs_PY79.featurized.bed) where each cigar operation had an associated coordinate in the recipient chromosome (X: mismatch, =: identical,

D: Deletion in donor, unique to recipient, I: Insertion in donor, unique to donor, N: Recipient base with no alignment to the donor). Bedtools intersect was then used to identify all overlapping cigar elements and subsequently trimmed to the queried genomic intervals using custom python code.

Window analysis of recombination breakpoints:

Recombination breakpoints were defined by the midpoint base of the crossover window for each site of transformation from the WT transformants. Recombination breakpoints that were associated with regions of homologous replacement or intervening sequences less than 1000 bp were omitted from the window analysis and analyzed separately to prevent overlapping windows between donor-derived and recipient-derived DNA. 1 kb flanking coordinates were constructed on either side of each breakpoint were obtained and split into non-overlapping 100 bp windows and annotated with their adjacent intervening recipient sequences. Windows that would extend beyond the coordinates of the genome were removed from the analysis. Percent identity of each window was conducted using the bedtools intersect of the featurized bed file discussed above.

Quantification and statistics:

A two-sample independent t-test was performed to identify potential significant differences between the mean GC content of short transfers (N=108 transfers) in the donor DNA transferred segments and the replaced recipient sequences using the Scipy stats module's ttest_ind (scipy.stats.ttest_ind) function. We further performed a one-sample t-test to identify if either of the donor or recipient GC content of the short transfers significantly differed from the genome(s) average GC content using the scipy.stats.ttest_1samp function. Pearson and Spearman correlations between the donor GC content and the length of the short transfers were performed using scipy.stats.pearsonr and scipy.stats.spearmanr functions.

Statistical tests to identify potential significant differences between the lengths of transferred segments according to their marker status were performed using a non-parametric Mann-WhitneyU test (scipy.stats.mannwhitneyu). Test statistics and number of transferred segments for each group are reported in the main text.

We also employed a one-way ANOVA (scipy.stats.f_oneway), with a follow-up Tukey-hsd multiple test correction (scipy.stats.tukey_hsd) to test whether the percent identity of the

transferred segments significantly differed across their marker grouping designations. The test statistics, p-values, and number of samples are reported in this file under **Figure S4D, E** and **Supplemental Table S1**.

Comparisons of lengths sites of transformation and their spacings between WT and Δ*mutS* were carried out using a non-parametric Mann-WhitneyU test using the scipy.stats.mannwhitneyu function. T-tests provided by the scipy.stats.ttest_ind function were used to test for differences between the counts of transferred patches and the percentage transformed within each cluster group between WT and Δ*mutS* transformants.

Linear mixed-effect models for each of the following response variables was carried out using the lmer4,lmerTest, and MuMIn libraries in R. Initial models were built for percent identity, GC content, and log10 median RPKM with the genotype (WT vs. *mutS*), genome label (donor vs recipient) and a log10 length bin, as categorical fixed effects, with a random effect from the cluster group (response variable ~ genotype*genome label*length bin + (1| cluster group).The interval lengths were treated as a categorical variable as the response variables did not scale linearly with length as a continuous explanatory variable. Since genotype was found to not be significant in any of the initial models, this term was dropped and refit using only the genome label and length bin (with interaction).

Two-way ANOVA tests concerning the percent identity within windows surrounding recombination breakpoints were carried out using the statsmodel library package with the ols() and anova_lm(type=3) functions.

Assumptions of normality were assessed for each case using a holistic approach, combining visualization of histograms, qqplots, a shapiro test for normality, and a random normal distribution under the same mean and standard deviation. Outputs for each of these can be found in the accompanying deposited data. Significance thresholds were defined as follows; ****: [0,0.0001], ***:(0.0001, 0.001], **(0.001, 0.01], *(0.01, 0.05], ns: (0.05, 1].

Supplemental Results

Mosaic transfer is a cross-phyla phenomenon

Given that the core natural transformation proteins are widely conserved (3), we hypothesized that mosaic transfers are likely to occur across distant phyla. We selected the naturally transformable, marine cyanobacteria *Picosynechoccocus* sp. PCC 7002 as a second model recipient strain. A parallel distributed marker approach was generated in the donor strain *Picosynechococcus* sp. PCC 11901, due to its high genome collinearity and an ANI that is sufficiently diverged from PCC 7002 for detecting recombination (ANI 96.32%). We simultaneously wished to rule out the possibility that the mosaic transfer observed in *Bacilli* was a technical artifact derived from the *in vitro* method to generate distributed markers throughout the donor DNA. For this purpose, from the distributed marker population, we selected an isolate that contained a single, stably integrated marker (NR001, FEK30_03020::*spcR*; obtained from Falbel et al. unpublished) to use as donor genomic DNA. Upon transformation of recipient *Picosynechococcus* sp. PCC 7002 with this donor genomic DNA, a representative transformant was sequenced, assembled and mapped against the *Picosynechococcus* sp. PCC 7002 reference genome (see methods for details). Patterns of mosaic transfer on either side of the insertion marker are visualized **(Figure S4)**. In summary, patterns of mosaicism during natural transformation are a cross phyla phenomenon, independent of the *in vitro* nature of distributed marker approaches.

Transferred segments exhibit similar features when controlling for size and marker status

We next considered whether selectable markers influence local or global transformation outcomes. Within a genome we therefore considered the relationship between sequence identity and lengths of transferred segments. Unless explicitly designated, the 2 kb *spcR*+ marker was omitted from the following percent identity calculations, as it is useful to contrast its contribution to the change in overall identity. As expected, the percent identity of the transferred segments is similar to the background average nucleotide identity of 90% for *B. vallismortis* DV1-F-3 and *B. subtilis* PY79 (**Figure S5A,B**). Yet, the distribution has a tail of lower percent identities, corresponding to predominately longer transferred segments, and includes all the transferred segments containing the *spcR* marker. Grouped according to their marker status, the lengths of transferred genomic regions that contained the marker (*spcR*+, N=11, range = 6,183-34,887 bp,

median=10,186 bp) were significantly longer than those that did not (*spcR-,*N=254, range=7-36,229 bp, median = 118 bp) (Mann-WhitneyU test-statistic=57.0, p-value=7.3e-08). However, the distributions of each marker group have considerable overlap, demonstrating that the lengths for *spcR*+ segments are well represented in the *spcR*- population (**Figure S5C**).

To better understand the influence of the presence of the marker on percent identity, we designed an expanded set of marker groupings that better controlled the length of the transferred segments between the two marker groups. Using the minimum transferred genomic length of the *spcR*+ group (6,183-34,887 bp) as a rationale guide, we split the *spcR*- group into two additional subgroupings according to those that were less than (*spcR*- 6 kb-, 7-5,960 bp), or greater than 6 kb (*spcR-,*6 kb+, 6,094-36,229 bp). Additionally, given that the marker itself is an inserted heterologous sequence, we further designated the *spcR*+ group into two additional subgroupings according to whether the marker was included in the percent identity calculation (*spcR*+ inclusive) or was excluded (*spcR*+ exclusive). For comparison, we include a background genome subgrouping, which is randomly sampled from the whole genome alignment between *B. vallismortis* and *B. subtilis* using the same length distribution as the *spcR*+ inclusive group.

We compared the BLAST-like percent identity for each of these marker groups using a one-way ANOVA (test-statistic=56.58, p-value=5.28e-35) with a follow-up Tukey-HSD test **(Figure S5D; Table S1).** The mean percent identity of the *spcR*- 6 kb-minus-marker group significantly differed from every other grouping (mean: 91.4, stdev: 3.95). In contrast, the mean percent identities of the *spcR*- 6 kb-plus-marker group (mean: 82.6, stdev 10.8) and the *spcR*+ exclusive (mean: 79.4, stdev: 15.1), did not significantly differ (p-value: 0.767). While the percent identities of *spcR* containing segments are biased towards lower identity, their identities do not differ significantly from those in a similar size range. This is further supported by the observation that the *spcR*+ 6 kb- and *spcR*+ exclusive marker groups do not differ significantly from a random sampling of the whole genome alignment (mean: 79.9, stdev: 15.27). Thus, the observed lowered percent identity of *spcR*+ segments is likely a function of the size of the interval extracted from the whole genome alignment itself, rather than the presence of a resistance cassette. As expected, when accounting for the ~2 kb nonhomologous *spcR* resistance cassette, the percent identities of the markerless groups under and over 6 kb in length do significantly differ from the *spcR*+ inclusive marker group (mean:67.5, stdev:11.79). To further explore the contribution of nonhomologous material to the percent identity of the transferred

segments we repeated the analysis with a gap-compressed calculation which compresses any continuous indels of the alignment into a single gap (matches / (mismatches + gap opens + matches)). No gap-compressed identity significantly differed across any marker grouping (one-way ANOVA statistic=0.403, p-value=0.806). We interpret these results to mean that longer transferred segments are more likely to include indels when compared to small, transferred segments.

Short transfers do not correlate with GC content

There was no significant difference between the mean donor (42.8%) and recipient (42.7%) GC content for transfer lengths under 100 bp (t-test statistic=1.42, p-value=0.16, df=214.0). Therefore, the GC content of the transferred donor DNA was chosen as a representative metric and is visualized for each transferred segment under 100 bp in **Figure 3**. Contrary to the MEPS-based association, no significant correlations were identified between the donor GC content and the length of the short transfers using a linear (Pearson correlation coefficient=-0.06, p-value=0.53) and rank-based spearman (Spearman correlation coefficient: -0.087, p-value: 0.37) approach. Likewise, the GC content of short transferred sequences under 100 bp were not significantly different from the average GC content of the recipient (t-test statistic=1.85, p-value=0.067, df=107, population mean= 43.8%) nor donor (t-test statistic=1.95, p-value=0.053, df=107, population mean=43.7%) genomes.

Supplemental References

1. Goris J, Konstantinidis KT, Klappenbach JA, Coenye T, Vandamme P, Tiedje JM. 2007. DNA-DNA hybridization values and their relationship to whole-genome sequence similarities. Int J Syst Evol Microbiol 57:81–91.

2. Boonstra M, Schaffer M, Sousa J, Morawska L, Holsappel S, Hildebrandt P, Sappa PK, Rath H, de Jong A, Lalk M, Mäder U, Völker U, Kuipers OP. 2020. Analyses of competent and non-competent subpopulations of Bacillus subtilis reveal yhfW, yhxC and ncRNAs as novel players in competence. Environ Microbiol 22:2312–2328.

3. Johnston C, Martin B, Fichant G, Polard P, Claverys J-P. 2014. Bacterial transformation: distribution, shared mechanisms and divergent control. Nat Rev Microbiol 12:181–196.

Supplemental Tables

Supplemental Table S1: P-values matrix for each of the Tukey-HSD pairwise comparisons for the BLAST-like identities displayed in Figure S5D corresponding to the groupings of marker vs. non-marker containing transferred segments (*spcR*+, *spcR*+), whether the length of the marker is included or excluded in the percent identity calculations (incl.,excl.) whether their lengths exceed or are less than 6 kb in length (6 kb+,6 kb-**).** The non-significant comparisons shown in **Figure S5D** are bolded.

	***spcR*- 6 kb-**	***spcR*- 6 kb+**	***spcR*+ excl.**	***spcR*+ incl.**	**background**
***spcR*- 6 kb-**	1.00	7.81e-05	2.33e-08	0.00	7.33e-08
***spcR*- 6 kb+**	7.81e-05	1.00	**0.767**	2.96e-07	**0.845**
***spcR*+ excl.**	2.33e-08	**0.767**	1.00	8.52e-05	**1.00**
***spcR*+ incl.**	0	2.96e-07	8.52e-05	1.00	4.37e-05
**background**	7.33e-08	**0.845**	**1.00**	4.37e-05	1.00

Supplemental Table S2: Type III ANOVA with Satterthwaite's method for the fit linear mixed effect model comparing percent identity (BLAST-like) between sites of transformation and their neighboring intervening recipient-derived sequences across clustered transfer events in the recipient chromosome.

**Category**	**Sum sq**	**Mean sq**	**NumDF**	**DenDF**	**F value**	**Pr(>F)**
Genome label	1.4	1.44	1	724.76	0.0161	0.8991
Length bin	4193.9	1398	3	733.69	15.597	7.64E-10
Genome label:Length bin	383.9	127.96	3	701.97	1.4276	0.2335

Supplemental Table S3: Type III ANOVA with Satterthwaite's method for the fit linear mixed effect model comparing GC content between sites of transformation and their neighboring intervening recipient-derived sequences across clustered transfer events in the recipient chromosome.

**Category**	**Sum sq**	**Mean sq**	**NumDF**	**DenDF**	**F value**	**Pr(>F)**
Genome label	271.62	271.62	1	730.2	4.4209	**0.03584***
Length bin	306.76	102.25	3	737.58	1.6643	1.73E-01
Genome label:Length_bin	696.83	232.28	3	719.84	3.7805	**0.0104***

Supplemental Table S4: Type III ANOVA with Satterthwaite's method for the fit linear mixed effect model comparing the log10 median gene expression between sites of transformation and their neighboring intervening recipient-derived sequences across clustered transfer events in the recipient chromosome.

**Category**	**Sum sq**	**Mean sq**	**NumDF**	**DenDF**	**F value**	**Pr(>F)**
Genome label	0.1949	0.1949	1	682.64	0.2829	0.595
Length bin	0.0422	0.0141	3	696.09	0.0204	9.96E-01
Genome label:Length_bin	0.6924	0.2308	3	727.95	0.335	0.8

Supplemental Table S5: Pairwise contrasts of GC across separate length bins using a Tukey-HSD multiple test correction and Kenward-Roger degrees of freedom method.

**contrast**	**Length bin**	**estimate**	**SE**	**df**	**t.ratio**	**p.value**
donor-recipient	0-10 bp	-4.706	3.66	742	-1.286	0.1989
donor-recipient	10-100 bp	-4.301	1.11	727	-3.859	**0.0001*****
donor-recipient	100-1000 bp	0.178	1.21	748	0.147	0.8829
donor-recipient	1000 bp+	0.249	0.948	739	0.263	0.7929

Supplemental Table S6: Two-way ANOVA of percent identities across 100 bp windows surrounding recombination breakpoints, with a minimum homologous replacement length of 1000 bp and a minimum of adjacent recipient DNA length of 1000 bp.

	**df**	**sum_sq**	**mean_sq**	**F**	**PR(>F)**
C(Window)	9	356	40	0.145777261	0.998350007
C(Genome label)	1	4675	4675	17.11226577	**3.70E-05*** **
C(window):C(Genome label)	9	836	93	0.339892958	0.961754563
Residual	1615	441174	273	NaN	NaN

Supplemental Table S7: Two-way ANOVA of percent identities across 100 bp windows surrounding recombination breakpoints, with a minimum homologous replacement length of 1000 bp and adjacent intervening recipient sequences falling between 1000 bp and the input DNA peak length of 51602 bp.

	**df**	**sum_sq**	**mean_sq**	**F**	**PR(>F)**
C(Window)	9	526	58	0.173757033	0.996605761
C(Genome label)	1	988	988	2.937540396	0.086886743
C(Window):C(Genome label)	9	234	26	0.077445352	0.999872869
Residual	900	302706	336	NaN	NaN

Supplemental Table S8: Two-way ANOVA of percent identities across 100 bp windows surrounding recombination breakpoints, with a minimum homologous replacement length of 1000 bp and adjacent intervening recipient sequences exceeding the input DNA peak length of 51602 bp.

	**df**	**sum_sq**	**mean_sq**	**F**	**PR(>F)**
C(Window)	9	891	99	0.520797592	0.860050566
C(Genome label)	1	4533	4533	23.85819036	**1.29E-06*** **
C(Window):C(Genome label)	9	1670	186	0.976491039	0.458090254
Residual	695	132048	190	NaN	NaN

Supplemental Table S9: Two-way ANOVA of percent identities across 100 bp windows surrounding recombination breakpoints, with a minimum homologous replacement length of 200 bp and a minimum of adjacent recipient DNA length of 200 bp.

	**df**	**sum_sq**	**mean_sq**	**F**	**PR(>F)**
C(window)	9	631	70	0.292965	0.976924672
C(side)	1	2853	2853	11.92352	**0.000561455*** **
C(window):C(side)	9	918	102	0.426542	0.921576765
Residual	3215	769152	239	NaN	NaN

Supplemental Table S10: Two-way ANOVA of percent identities across 100 bp windows surrounding recombination breakpoints, with a minimum homologous replacement length of 200 bp and adjacent intervening recipient sequences falling between 200 bp and the input DNA peak length of 51602 bp.

	**df**	**sum_sq**	**mean_sq**	**F**	**PR(>F)**
C(window)	9	794	88	0.413443	0.928631271
C(side)	1	75	75	0.350243	0.55403831
C(window):C(side)	9	356	40	0.185589	0.995654562
Residual	2140	456679	213	NaN	NaN

Supplemental Table S11: Two-way ANOVA of percent identities across 100 bp windows surrounding recombination breakpoints, with a minimum homologous replacement length of 200 bp and adjacent intervening recipient sequences exceeding the input DNA peak length of 51602 bp.

	**df**	**sum_sq**	**mean_sq**	**F**	**PR(>F)**
C(window)	9	244	27	0.093151	0.999725114
C(side)	1	6468	6468	22.22395	**2.75E-06*** **
C(window):C(side)	9	1743	194	0.665566	0.740624428
Residual	1055	307023	291	NaN	NaN

Supplemental Figures

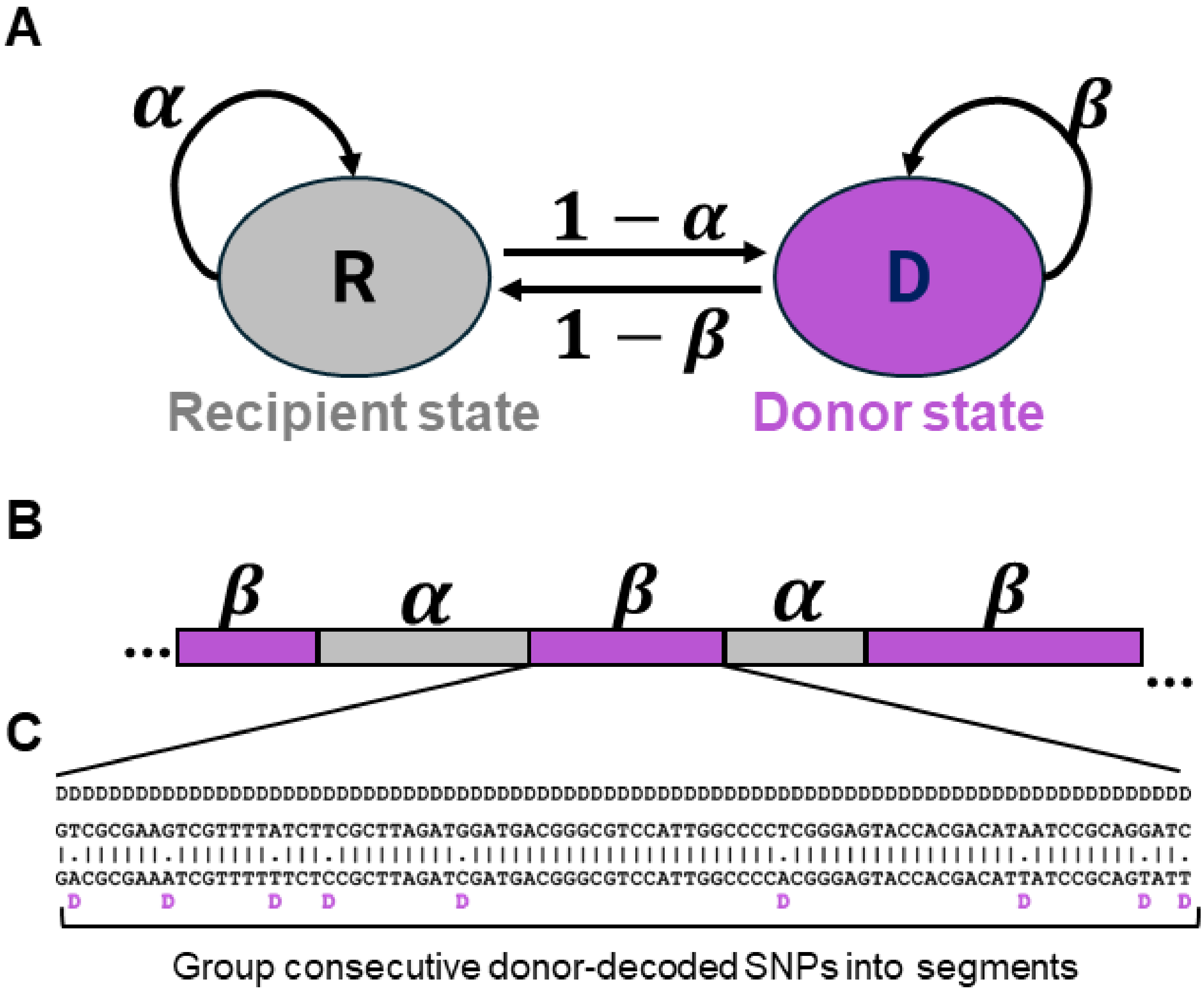

<u>Figure S1:</u> An HMM for the identification of mosaic patterns of transformation. A. A state transition diagram highlighting the two possible genomic states (recipient or donor) across every position of the recipient genome. α encodes the probability that the next base will not be recombined in the recipient genome, and β denotes the probability that the next base will continue in a recombination event from the donor. **B.** A conceptual diagram depicting what lengths the α and β parameters influence. The β parameter governs the length of recombined donor segments (purple patches), whereas the α parameter dictates the lengths of the recipient sequences between donor recombined segments (gray patches). **C.** A conceptual depiction of the assignment of recombined segments. The topmost row corresponds to the decoded Viterbi path assignments across each of the positions in the recipient genome, with the run of successive D characters corresponding to predicted donor alleles. Recombined segments were assigned by grouping consecutive donor state predictions at the sites of orthologous SNPs.

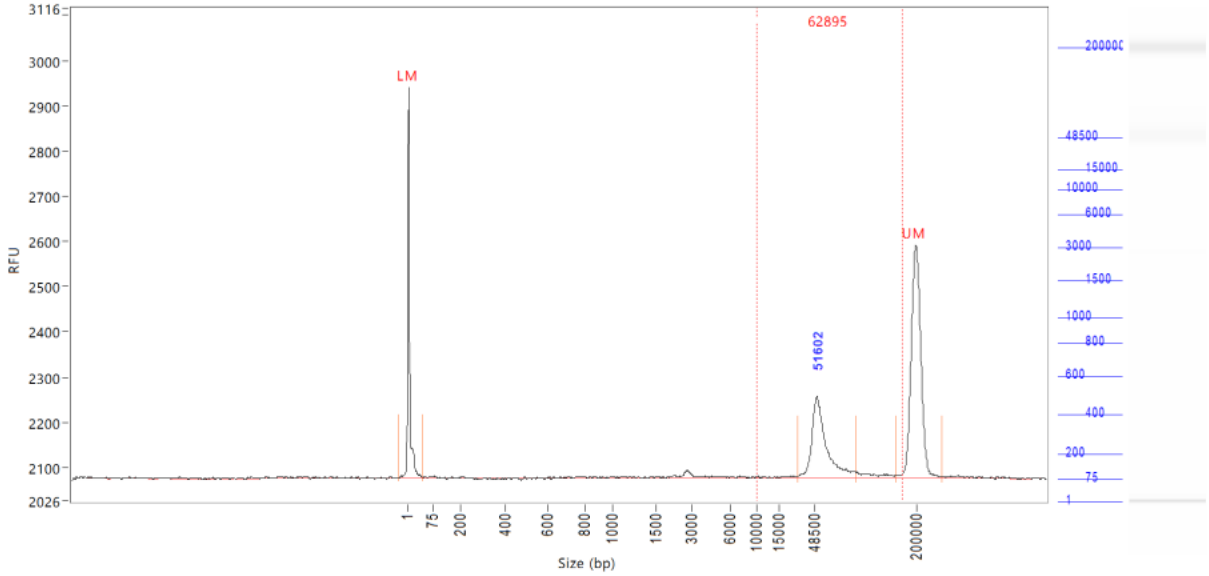

Peak	Size (bp)	Conc. (ng/uL)	From (bp)	To (bp)	Avg. Size (bp)	CV%	RFU	Corr. Peak Area
1	1 (LM)	0.0142	0	43	2	322.49	861	5.518
2	51602	0.1159	32719	110961	58901	24.63	179	3.748
3	200000 (UM)	0.0176	170763	239844	200033	3.53	512	6.814

TIC:	0.1159	ng/uL
TIM:	0.0032	nmole/L
Total Conc.:	0.1259	ng/uL

Smear Analysis 10000 bp to 180000 bp 0.1223 ng/ul 97.1 %Total 0.0032 nmole/L 62895 Avg. Size (b.p.) 36.76 %CV

Figure S2: **Genomic DNA isolated from *B. vallismortis* corresponds to high molecular weight DNA.** A bioanalyzer gel-electrophoresis analysis of the input *B. vallismortis* DNA, with a smear analysis highlighting the mean size of the input DNA species from 10 kb to 180 kb, as well as the peak height of the most abundant DNA length shown at 51602 bp. LM and UM peaks correspond to lower molecular weight and upper molecular markers respectively for normalization and quantification to a known standard.

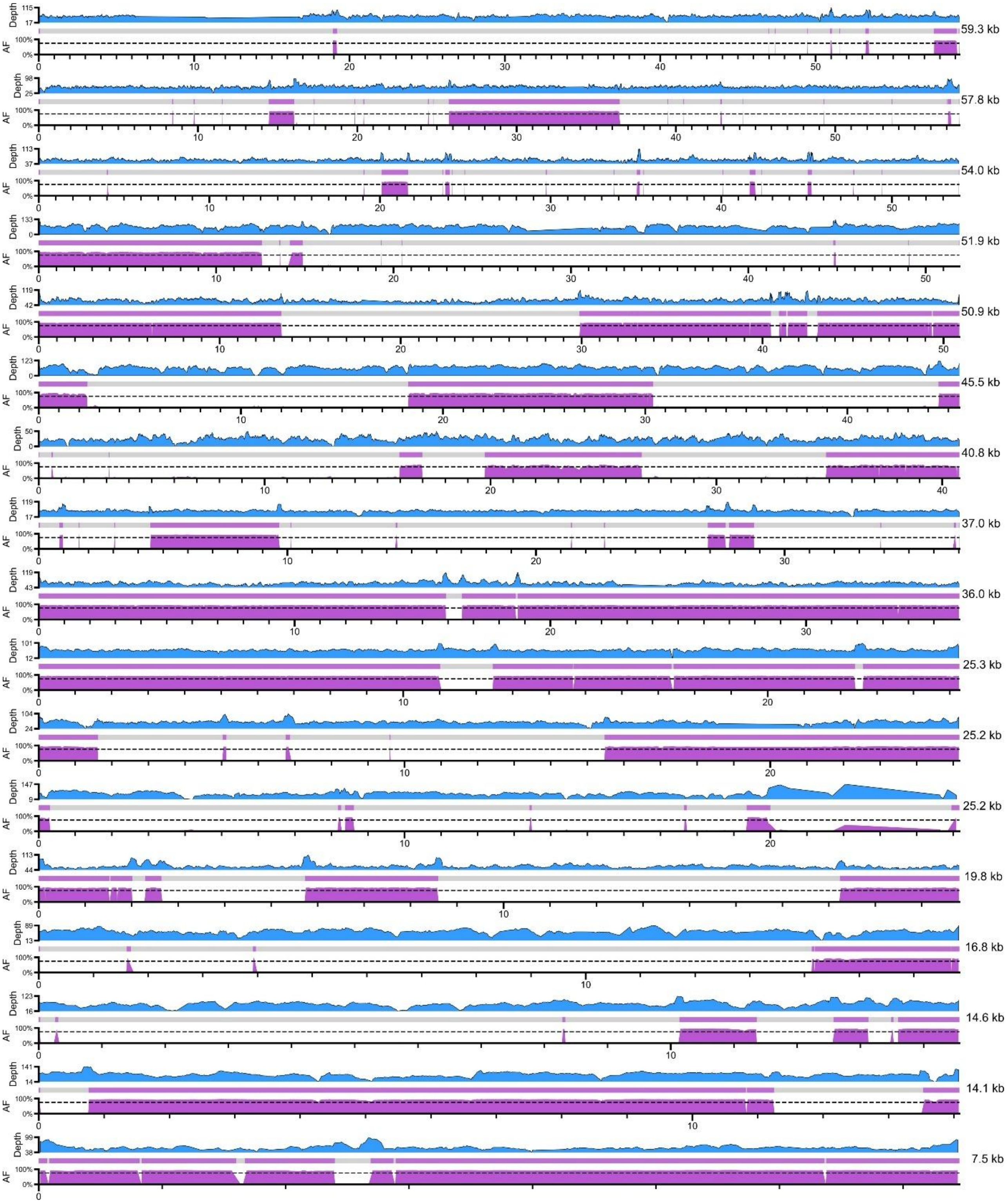

<u>Figure S3:</u> MHMM predictions are consistent with the observed donor allele frequencies at SNP positions. A confidence plot for each cluster group shown in Figure 2E. For each cluster group the coverage in the down-sampled VCF files is displayed as a density plot (top axis, blue fill), along with the donor allele frequency (bottom-most axis, purple fill), with a visual aid of 75% as a horizontal, dashed line. Each minor tick on the x-axis corresponds to 1 kb.

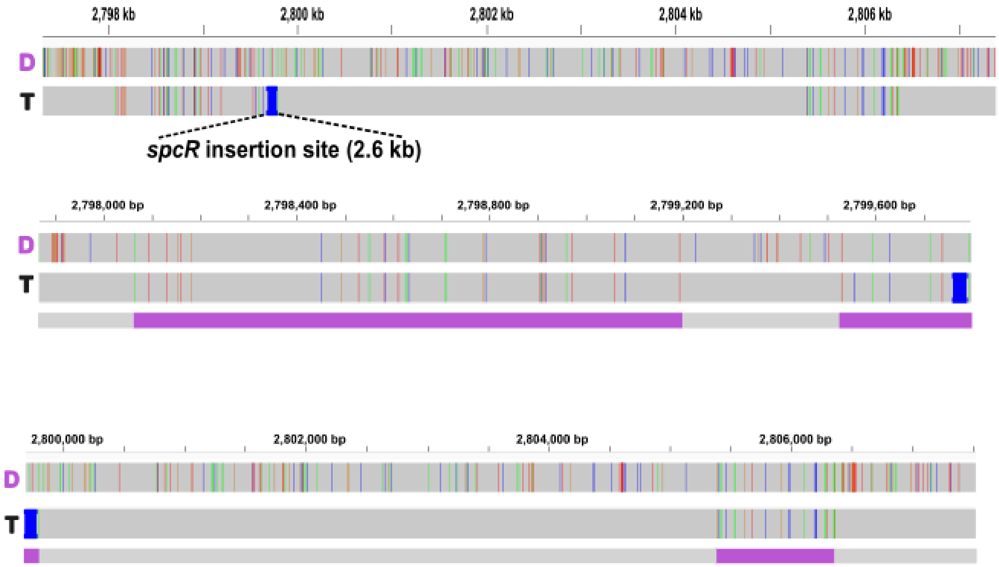

Figure S4: Mosaic sites of transformation are a cross-phyla phenomenon in the cyanobacteria *Picosynechococcus*. IGV representation of the assembled transformant genome mapped against the recipient genome *Picosynechococcus* sp. PCC 7002. The upper IGV contig (D) corresponds to the orthologous donor *Picosynechococcus* sp. PCC 11901 sequence, highlighting the locations of donors distinguishing SNPs between the two genomes. The lower IGV contig (T) displays the presence or absence of donor-distinguishing SNPs in the assembled *Picosynechococcus* sp. PCC 7002 genome. The ~2.6 kb *spcR* marker derived from transforming with gDNA from *Picosynechococcus* sp. PCC 11901 FEK30_03020::*spcR* is shown as a blue rectangle. The uppermost IGV snapshot corresponds to a "birds eye view" of the local regions surrounding the site of transformation. The middle snapshot highlights sites of mosaic transformation to the left (5') end of the inserted marker. The lowermost snapshot corresponds to the right (3') end of the inserted marker.

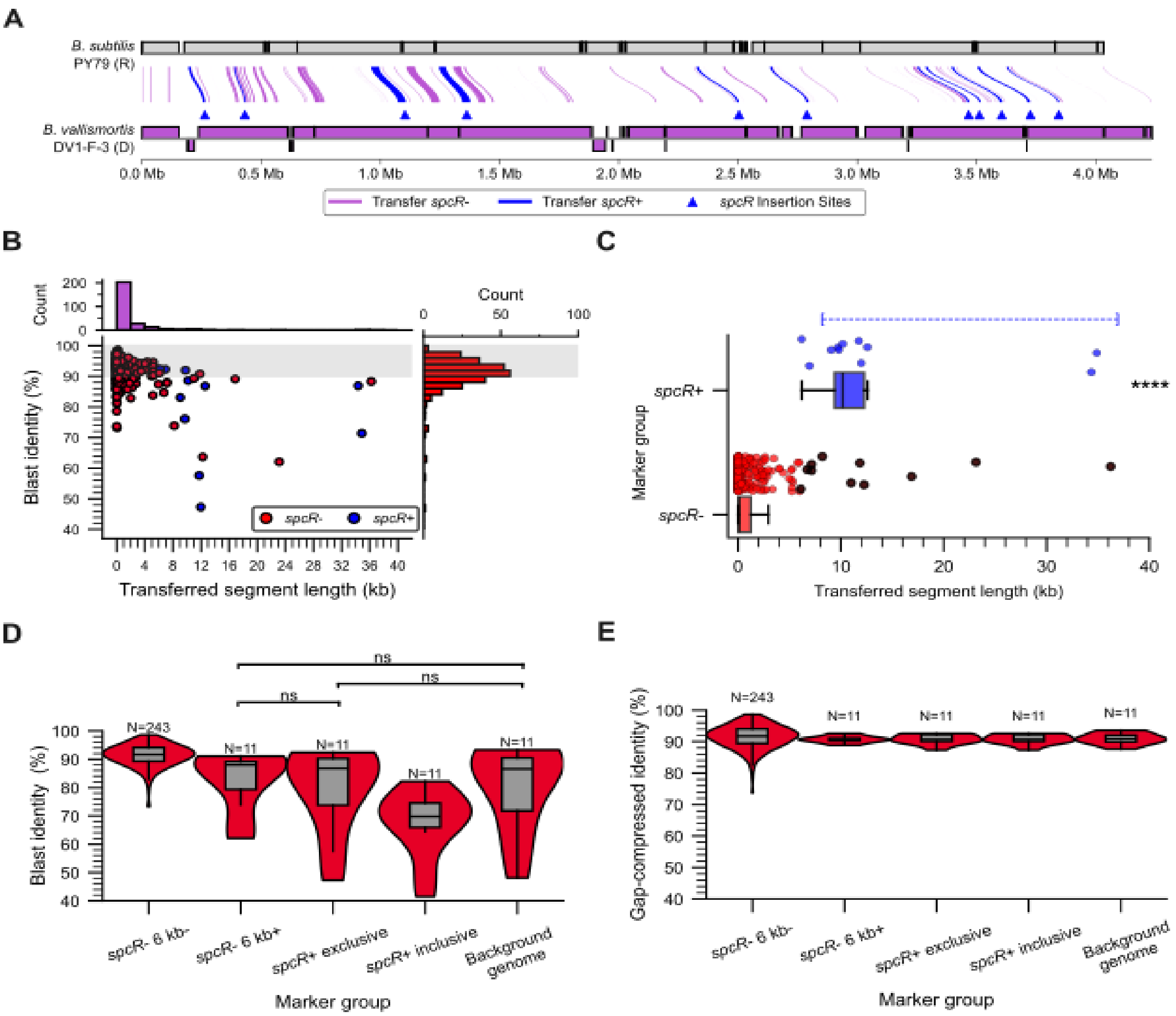

<u>Figure S5:</u> Distributed markers are biased towards longer transfer lengths. A. Linear genome diagrams of the collinear blocks between the naturally transformable recipient, *B. subtilis* PY79 (top), and the donor *B. vallismortis* DV1-F-3 (bottom). Chords between the genomes correspond to sites of transfer containing the *spcR* marker (*spcR*+, blue) and those that do not (*spcR*-, purple). **B.** A marginal histogram highlighting the relationship between the transferred segment length and their respective percent identity for *spcR*- (red) and *spcR*+ (blue). **C.** Lengths of transferred segments according to their marker status *spcR*- (red) and *spcR*+ (blue) are shown in a boxplot and accompanying whisker plot format. The *spcR*+ plot corresponds to the lengths of the donor chromosome that was transferred while excluding the length of the *spcR* marker. To account for the true length of the transferred segment, a blue, dashed whisker plot is shown for the shifted range of values when the marker is included in the length calculations. Black scatter points in the *spcR*- group specifically highlight the 11 segments that exceed 6 kb, which are separated in the remaining plots D-E. **D.** Violin boxplots for BLAST-like identities of the transferred segments according to their marker status and size range. Due to the large number of significant differences, only the non-significant results are highlighted, and the omitted significant pairwise comparisons are reported in (**Table S1). E.** The gap-compressed percent identities for the same marker groups displayed in D.

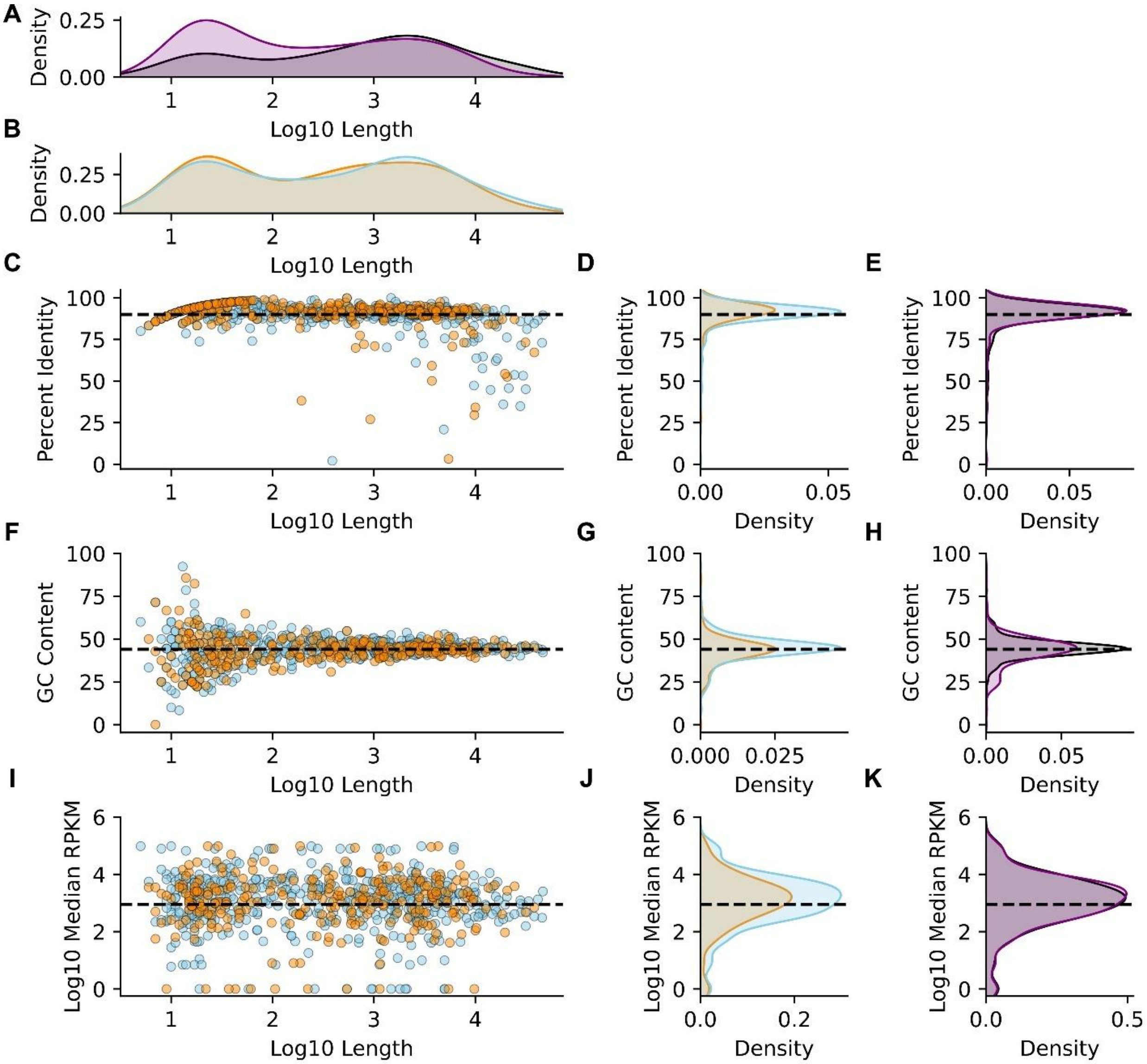

<u>Figure S6:</u> Distributions of sequence level features do not largely differ by genotype or genome of origin across mosaic clusters. **A.** Kernel density estimates for the lengths of genomic intervals derived from donor (purple) and recipient (dark grey) intervals across sites of clustered mosaic exchange. **B.** Kernel density estimates same data as A, but our colored with respect to WT (light blue) or Δ*mutS* (orange) genotypes of the transformants. **C-E.** Scatterplot of the percent identity as a function of the length of the genomic interval (C), with kernel density estimates of the distributions by genotype (D) and by genome of origin (E). **F-H.** Same schematic as C-E but for the GC content of the genomic intervals**. I-K.** Same schematic as **C-H** but for the log10 median RPKM values across genes that overlap the genomic intervals.

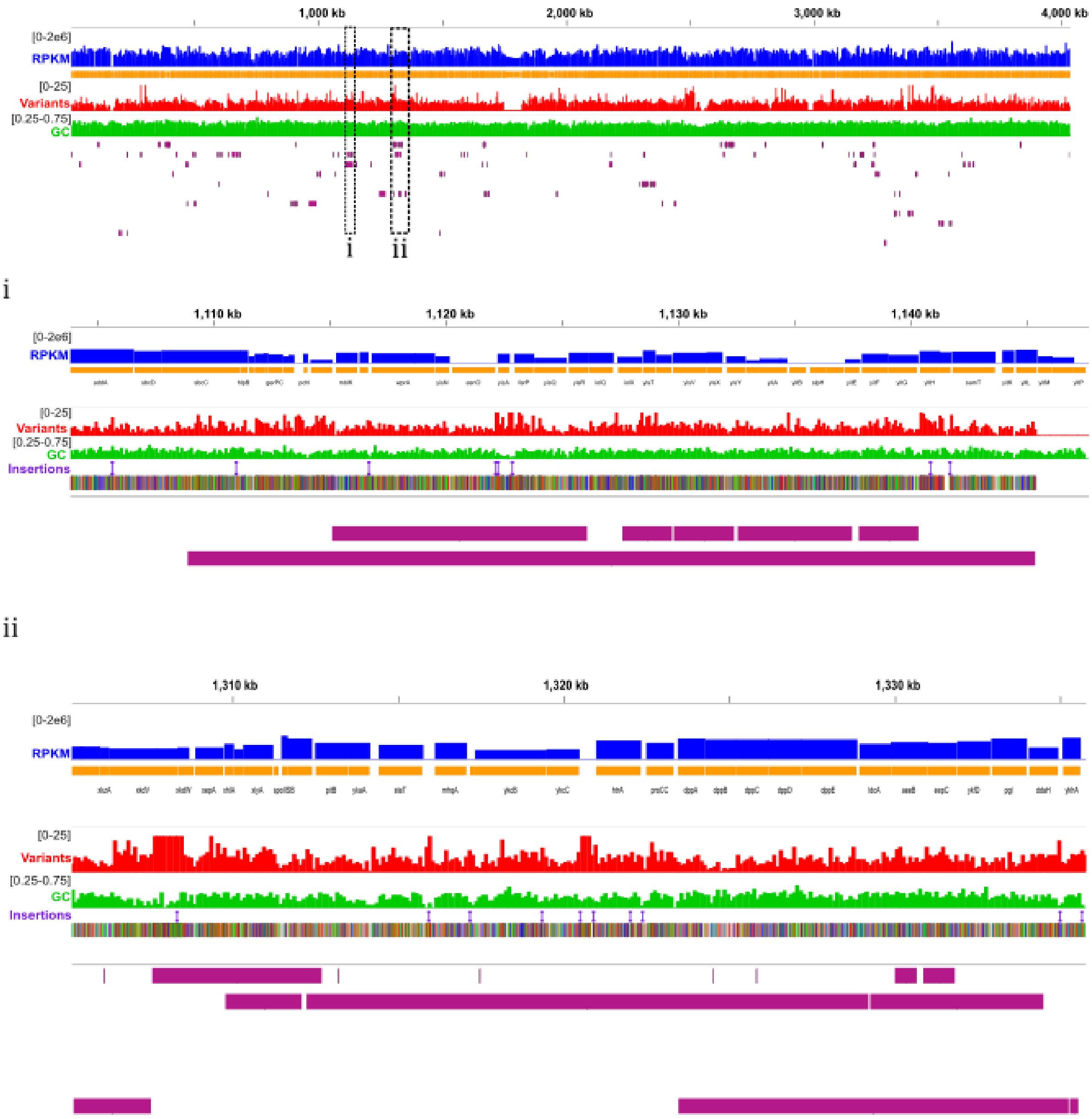

Figure S7: **Sites of informative mosaic exchange lack local explanatory factors.** A pileup of genomic features across the 11 transformants are visualized in IGV, including zoomed regions of interest for overlapping sites of mosaic transfer across more than one transformant sample. The description for each track is described here in order, beginning at the topmost track: average RPKM of genes expressed in the competent cell state plotted on a $\log_{10}$ scale (blue bar chart); counts of sequence variants (red bar chart) across a sliding window (window and step size of 100 bp); percent GC of the recipient genomic regions (green bar char) across a sliding window (window and step size of 100 bp); locations of insertions (purple Is); aligned donor DNA genome sequence derived from the progressiveMauve LCBs; remaining tracks correspond to sites of homologous replacement in the recipient genome (purple rectangles), with each track representing a separate transformant.

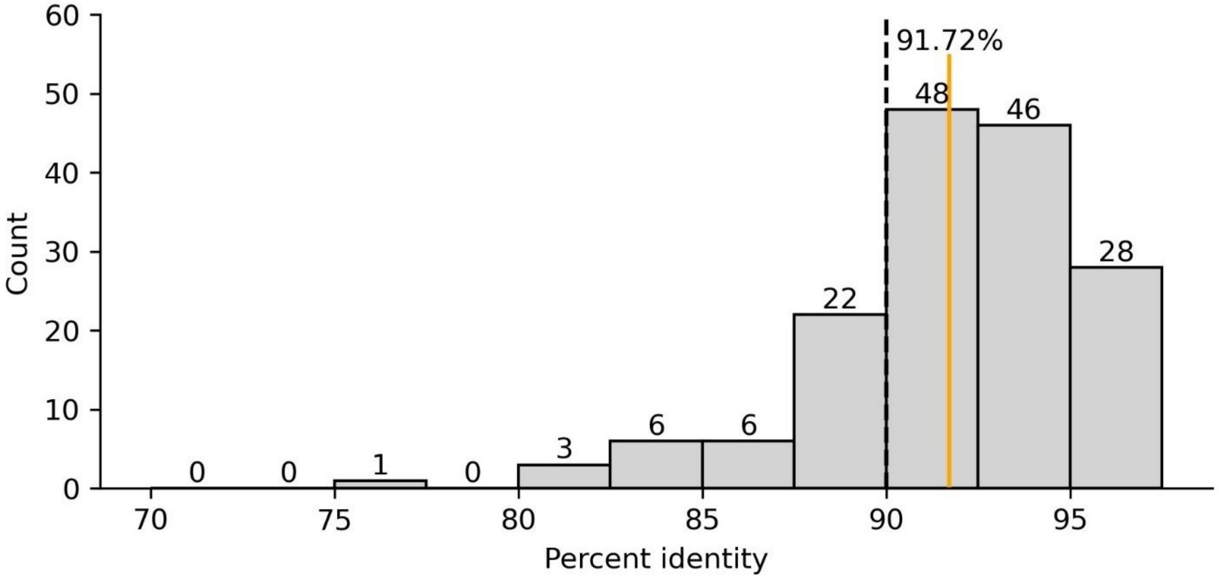

Figure S8: Short intervening recipient sequences exceed the average nucleotide identity. Histogram of the percent identities of short intervening recipient segments (less than 1000 bp) adjacent to sites of transformation in the recipient chromosome. The dashed, black, vertical line denotes the 90% average nucleotide identity between the donor and recipient genome. The vertical orange line depicts the average percent identity of the short intervening recipient sequences (91.72%).
