# Supplementary material for: High-resolution genomic analysis reveals abundant mosaic outcomes of bacterial natural transformation independent of MutS-mediated mismatch repair
Source: mBio. 2026 Jun 15;17(7):e00444-26. doi: 10.1128/mbio.00444-26 (PMC13344027; doi:10.1128/mbio.00444-26)
Supplement: Supplemental Material Descriptions — Descriptions for each supplemental file. [file mbio.00444-26-s0007.pdf]

Supplemental Information

Supplemental File S1: A PDF document containing extended bioinformatic methods, supplemental table S1, supplemental figures S1-S7.

Supplemental File S2: An excel (xlsx) file containing the locations of the 9 bp duplication (insertion site) of the *spcR* marker across each of the eleven WT transformants.

Supplemental File S3: A TSV dataframe of the transferred segments defining the start and end by the innermost donor SNP alleles for WT samples.

Supplemental File S4: A TSV dataframe of the transferred segments defining the start and end by the midpoint of the crossover window for WT samples.

Supplemental File S5: A TSV dataframe of the transferred segments defining the start and end by the outermost base of the crossover window (up to but not including the recipient-defining alleles) for WT samples.

Supplemental File S6: A TSV dataframe containing WT and MutS data used in the analysis.
